# Induction and Maintenance of CX3CR1-Intermediate Peripheral Memory CD8^+^ T Cells by Persistent Viruses and Vaccines

**DOI:** 10.1016/j.celrep.2018.03.074

**Published:** 2018-04-17

**Authors:** Claire Louse Gordon, Lian Ni Lee, Leo Swadling, Claire Hutchings, Madeleine Zinser, Andrew John Highton, Stefania Capone, Antonella Folgori, Eleanor Barnes, Paul Klenerman

**Affiliations:** 1 Peter Medawar Building for Pathogen Research, University of Oxford, Oxford OX2 3SY, UK; 2 Reithera SRL (formerly Okairos SRL), Viale Città d’Europa 679, 00144 Rome, Italy

**Keywords:** cytomegalovirus, T cells, memory, adenovirus, vaccination, CX3CR1, memory inflation, mouse, human

## Abstract

The induction and maintenance of T cell memory is critical to the success of vaccines. A recently described subset of memory CD8^+^ T cells defined by intermediate expression of the chemokine receptor CX3CR1 was shown to have self-renewal, proliferative, and tissue-surveillance properties relevant to vaccine-induced memory. We tracked these cells when memory is sustained at high levels: memory inflation induced by cytomegalovirus (CMV) and adenovirus-vectored vaccines. In mice, both CMV and vaccine-induced inflationary T cells showed sustained high levels of CX3R1^int^ cells exhibiting an effector-memory phenotype, characteristic of inflationary pools, in early memory. In humans, CX3CR1^int^ CD8^+^ T cells were strongly induced following adenovirus-vectored vaccination for hepatitis C virus (HCV) (ChAd3-NSmut) and during natural CMV infection and were associated with a memory phenotype similar to that in mice. These data indicate that CX3CR1^int^ cells form an important component of the memory pool in response to persistent viruses and vaccines in both mice and humans.

## Highlights

• CX3CR1^int^ T cells are a large component of memory induced by CMV and adenovaccines
• Inflating CX3CR1^int^ cells exhibit an effector-memory phenotype but can proliferate
• Human inflationary CX3CR1^int^ cells induced by CMV and adenovaccines share features
• CX3CR1^int^ cells contribute to memory expansion in specific infections or vaccines

## Graphical Abstract

**Figure:**
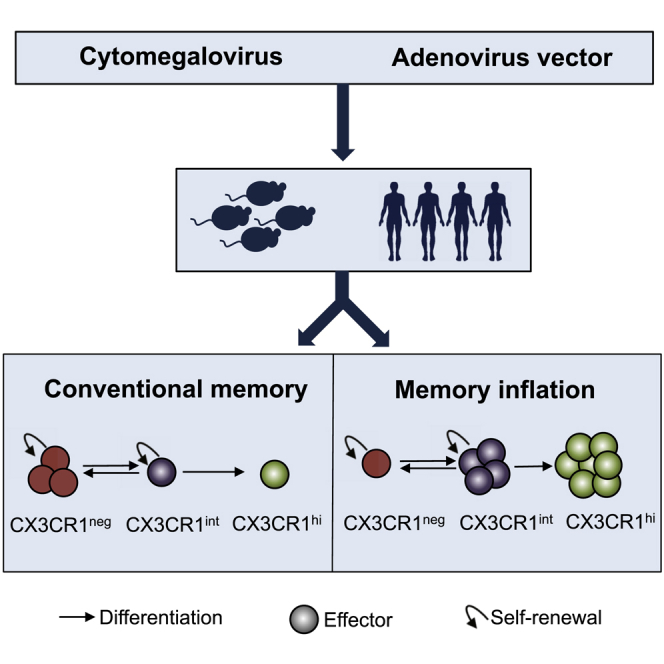

## Teaser

Gordon et al. demonstrate that CX3CR1^int^ peripheral memory T cells are a substantial component of memory inflation induced by persistent CMVs and adenoviral vaccination. They are characterized by sustained proliferation and an effector-memory phenotype linked to these expanded CD8+ T cell memory responses. Core phenotypic features are shared by humans and mice.

## Introduction

During an infection, naive T cells become activated and proliferate upon recognition of cognate antigen, giving rise to effector subsets. While effector T cell populations contract following control of infection, a fraction persists as diverse long-lived memory T (Tmem) cells. Tmem cells are heterogeneous subsets defined by homing receptor expression and tissue localization. Traditionally, two major Tmem subsets in blood were defined based on expression of lymph node homing receptors CCR7 and CD62L, denoting CCR7^+^/CD62L^+^ central-memory (TCM) cells, with the capacity to migrate to lymphoid tissues, and CCR7^−^/CD62L^−^ effector-memory (TEM) cells, which are able to migrate to peripheral tissues (Sallusto et al., 1999). TCM cells have superior ability to produce interleukin-2 (IL-2), proliferate and persist within the host, and become the predominant memory CD8^+^ T cell subset over time (Wherry et al., 2003). By comparison, TEM cells are more cytotoxic, migrate to diverse peripheral tissues sites, and until recently, were thought to be the dominant subset performing tissue surveillance (Sallusto et al., 1999). Recently identified, non-migratory tissue-resident memory (TRM) cells, a third Tmem subset, are localized in diverse tissue sites and mediate local protective immune responses (Mueller and Mackay, 2016).

Gerlach et al. (2016) identified a fourth Tmem subset, peripheral memory (TPM) cells, which are defined by intermediate levels of expression of the chemokine receptor CX3CR1 (Fractalkine receptor). Using *Cx3cr1*^*+/gfp+*^ reporter mice infected with lymphocytic choriomeningitis virus (LCMV) or vaccinia virus, Gerlach et al. (2016) showed that CX3CR1^int^ TPM cells, rather than CX3CR1^hi^ TEM cells, are the predominant migratory Tmem cells that survey peripheral tissues. In addition, CX3CR1^int^ TPM cells have superior homeostatic proliferation capacity compared with other Tmem subsets and not only self-renew but also contribute to the expanding CX3CR1^neg^ TCM pool. The identification of CX3CR1^int^ TPM cells necessitates re-examination of the roles that different CD8^+^ T cell subsets play in immunosurveillance and protection against re-infection to a variety of pathogens.

CD8^+^ T cells are crucial for the immune response to infections and cancer, and eliciting large and persistent effector T cell populations has been the focus of vaccine development. The role of CX3CR1^int^ TPM cells in the generation and maintenance of robust vaccine-derived Tmem populations is yet to be fully explored. Cytomegalovirus (CMV) and adenoviral vectors induce an expanded, sustained TEM CD8^+^ T cell response to specific epitopes, termed memory inflation, leading to interest in these cells as vaccine modalities (Klenerman and Oxenius, 2016). Inflationary T cells maintain effector function and ability to proliferate but lack features of T cell exhaustion (Klenerman and Oxenius, 2016). CMV infection in mice and humans (MCMV and HCMV, respectively) can lead to single-specificity inflationary T cells comprising of up to 20% of the circulating T cell pool, which develop in parallel with conventional non-inflating TCM responses to many epitopes (Karrer et al., 2003). Preclinical models of HIV vaccines using simian CMV vectors show promise and generate atypical major histocompatibility complex (MHC) class II and human leukocyte antigen (HLA)-E-restricted CD8^+^ T cell responses (Hansen et al., 2011, Hansen et al., 2016). Adenoviral vector-induced T cell responses in a murine model using a recombinant replication-deficient human adenovirus 5 (HuAd5) vector expressing β-galactosidase (Ad-lacZ) led to memory inflation of T cell responses to one of two immunodominant epitopes (Bolinger et al., 2013). Clinical studies of replication-deficient adenoviral vectors have shown potency in generation of antiviral T cell pools with features that overlap with those of inflated populations in MCMV and HCMV infection (Bolinger et al., 2015, Swadling et al., 2014).

Understanding induction and maintenance of robust T cell memory is important for the development of CD8^+^ T cells vaccines that aim to induce large numbers of memory CD8^+^ T cells of a favorable phenotype able to provide optimal protection against complex pathogens. However, the phenotype of the memory cells that maintain large CD8^+^ T memory pools remains incompletely defined (Klenerman and Oxenius, 2016). The identification of CX3CR1^int^ TPM cells with enhanced self-renewal and proliferative properties (Gerlach et al., 2016) prompted us to explore the role of CX3CR1^int^ TPM cells in the generation and maintenance of inflating and conventional Tmem populations induced by persistent infection and non-replicative adenoviral vectors in mice and humans. In mouse models, both CMV and vaccine-induced inflationary CD8^+^ T cells showed high frequencies of CX3CR1^int^ cells exhibiting a TEM phenotype but delayed differentiation, in the early memory phase, compared to conventional CD8^+^ T cell memory. CX3CR1 expression was not required for memory inflation, although blunted memory cell frequencies and differentiation were seen in *Cx3cr1*^*gfp+/gfp+*^ mice following vaccination. As in mice, humans receiving an adenovirus-vectored vaccine for hepatitis C virus (HCV) (ChAd3-NSmut) had CX3CR1^int^ CD8^+^ T cells that were strongly induced and maintained in the long term and were associated with a TEM phenotype. Similar observations were made in natural HCMV infection. These data indicate that CX3CR1^int^ memory cells form a substantial component of the memory pool in response to persistent viruses and vaccines in both mice and humans.

## Results

### MCMV Infection Induces Three Subsets Based on CX3CR1 Expression Levels in Conventional and Inflating Virus-Specific CD8^+^ Tmem Cells

**Figure 1:**
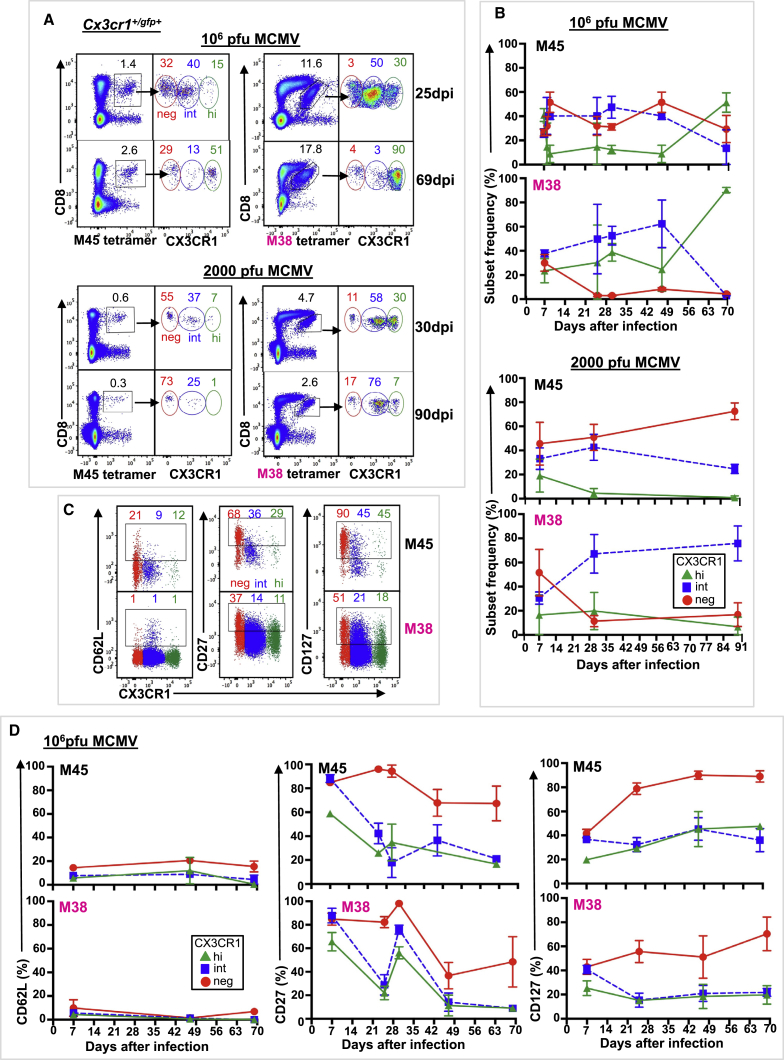
Persistent Infection with Murine CMV Induces Three Distinct Populations Based on CX3CR1 Expression in Conventional and Inflationary CD8^+^ T Cell Memory *Cx3cr1*^*+/gfp+*^ mice were infected i.v. with 10^6^ PFU (full dose) or 2,000 PFU (low dose) MCMV, and blood was serially sampled. Conventional memory responses (black label) were assessed by M45-tetramer staining, and inflationary memory responses (pink label) were assessed by M38-tetramer staining. neg, red; int, blue; hi, green. Coloring is consistent for all panels. (A) Composite fluorescence-activated cell sorting (FACS) plots (n = 2–5) of M45- and M38-tetramer staining of live lymphocytes (left) and CX3CR1 subsets of tetramer^+^ CD8^+^ T cells (right) early (25 or 30 days post-infection [dpi]) and late (69 or 90 dpi). Mean tetramer^+^ CD8^+^ T cells and mean CX3CR1 subsets are indicated (n = 2–5). (B) Mean (±SD) CX3CR1 subsets of M45- and M38-tetramer^+^ CD8^+^ T cells (n = 2–9). (C and D) Expression of surface markers CD62L, CD27, and CD127 by CX3CR1 subset on M45- and M38-tetramer^+^ CD8^+^ T cells. (C) Composite FACS plots (n = 3) from left to right (L to R) of CD62L, CD27, and CD127 expression on M45- and M38-tetramer^+^ CD8^+^ T cells 47 dpi, showing mean expression for each CX3CR1 subset (n = 3). (D) Mean (±SD) expression (L to R) of CD62L, CD27, and CD127 by CX3CR1 subset on M45- and M38-tetramer^+^ CD8^+^ T cells (n = 2–3). Data are compiled from 2–5 mice from 2–4 independent time course experiments. See also Figures S1 and S2.

To characterize CX3CR1 expression on CD8^+^ Tmem cells in persistent viral infection, we first analyzed the well-characterized model of MCMV infection. Intravenous (i.v.) infection of C57BL/6 mice with 10^6^ plaque-forming unit (PFU) MCMV results in two distinct CD8^+^ T cell memory (Tmem) responses, the conventional (contracting) and the expanded (inflating) CD8^+^ T cell response in blood (Figure S1) (Bolinger et al., 2015). Conventional memory responses in the MCMV model provide an internal control for analysis of the inflating memory response, because variables such as viral replication and antigen persistence are identical. CX3CR1 expression levels distinguished three virus-specific CD8^+^ Tmem subsets (CX3CR1^neg^, CX3CR1^int^, and CX3CR1^hi^) in both conventional (M45-tetramer^+^) and inflating (M38-tetramer^+^) responses (Figures S2A–S2D; Table S3), while uninfected C57BL/6 mice were devoid of CD8^+^ T cells expressing CX3CR1 (Figure S2A). To further characterize CX3CR1 expression, we used *Cx3cr1*^*+/gfp+*^ reporter mice in which GFP was introduced into the *Cx3cr1* locus. Following i.v. injection of 10^6^ PFU (high dose) MCMV, three subsets based on CX3CR1 expression levels were distinguished in conventional and inflating CD8^+^ Tmem responses in blood (Figure 1A). GFP-expressing CD8^+^ T cells were absent from uninfected *Cx3cr1*^*+/gfp+*^ mice (Figure S2A) (Böttcher et al., 2015, Gerlach et al., 2016). The frequency distribution of MCMV-specific CX3CR1^int^ and CX3CR1^hi^ Tmem cells differed in C57BL/6 mice compared to *Cx3cr1*^*+/gfp+*^ mice (Figure S2C), likely reflecting the transient surface expression of CX3CR1 on C57BL/6 mice and the sustained fluorescence of GFP (half-life > 24 hr), which is unaffected by CX3CR1 internalization (Jung et al., 2000).

During persistent MCMV infection, conventional Tmem cells maintained substantial frequencies of CX3CR1^neg^ cells (Figures 1A and 1B). Conventional Tmem populations maintained high mean frequencies of CX3CR1^int^ cells (40%–47%) in the early memory phase (21–49 days post-infection [dpi]) before CX3CR1^neg^ and CX3CR1^hi^ cells (58% and 51%, respectively) become more frequent in the late memory phase (≥50 dpi) (Figure 1B). Frequencies of CX3CR1^int^ Tmem cells following acute viral infection are markedly lower (∼15%) (Gerlach et al., 2016). To address whether the infective dose influences CX3CR1 expression, we assessed CX3CR1 expression in *Cx3cr1*^*+/gfp+*^ mice infected i.v. with 2,000 PFU (low dose) MCMV (Figures 1A and 1B). Again, conventional Tmem populations maintained high mean frequencies of CX3CR1^int^ cells (24%–44%), and Tmem switched to predominantly CX3CR1^neg^ cells at later memory time points, in keeping with conventional memory responses following acute viral infection (Gerlach et al., 2016).

In comparison, inflationary Tmem cells during persistent MCMV infection were nearly all CX3CR1 positive (int and hi) (Figures 1A and 1B), consistent with previous reports (Bolinger et al., 2015). Similar to the conventional Tmem response, inflationary Tmem populations maintained high mean frequencies of CX3CR1^int^ cells (50%–62%) in the early memory phase (21–49 dpi) before switching to predominantly CX3CR1^hi^ cells (90%) in the late memory phase (≥50 dpi) (Figure 1B). Following infection with low-dose MCMV, inflationary Tmem populations again maintained high mean frequencies of CX3CR1^int^ cells (67%–75%); however, Tmem did not switch to CX3CR1^hi^ cells at later time points (Figures 1A and 1B). Altogether, these results suggested that low-level viral replication supports CX3CR1^int^ Tmem cells, while high-level viral replication drives CX3CR1^hi^ Tmem cells in both conventional and inflating Tmem populations.

### CX3CR1^int^ CD8^+^ T Cells Retain a TEM Phenotype during Memory Inflation

We next asked whether tissue homing, activation state, or maintenance profile differed among CX3CR1 subsets in Tmem cells in blood following MCMV infection. We examined lymph node homing based on L-selectin (CD62L), differentiation based on CD27, and the homeostatic cytokine interleukin-7 receptor (IL-7R) CD127. CD27 is upregulated during the first days after T cell receptor activation and downregulated during T cell effector differentiation (Hintzen et al., 1993). CD127 expression is downregulated by activated effector T cells (Kiazyk and Fowke, 2008) and on inflationary CD8^+^ T cells following response to IL-7 (Bolinger et al., 2013). Previous study of Tmem cells using acute viral infection models (Gerlach et al., 2016) have described CX3CR1^neg^ cells as TCM (CD62L^+^, CD27^+^, CD127^+^), CX3CR1^hi^ cells as TEM (CD62L^−^, CD27^−^, CD127^−^), and CX3CR1^int^ cells as having phenotypic features more similar to CX3CR1^neg^ cells (CD62L^+/−^, CD27^+^, CD127^+^). In contrast, persistent MCMV infection led to a CX3CR1^int^ phenotype that closely resembled TEM CX3CR1^hi^ cells in both conventional and inflating Tmem populations in *Cx3cr1*^*+/gfp+*^ (Figures 1C and 1D) and wild-type (WT) C57BL/6 mice (Figures S2E and S2F). CD62L expression remained low on CX3CR1^int^ cells regardless of infective dose (Figure 1D; Figure S2I), and CD27 and CD127 expression on CX3CR1^int^ cells decreased over time (Figures 1C and 1D; Figures S2E and S2F). Our results suggest that in persistent MCMV infection, the overall phenotype of CX3CR1^int^ cells is that of an TEM pool, reflecting the well-described features associated with this setting of persistent infection and antigen exposure. Nevertheless, even within the TEM pool, they showed some delayed differentiation compared to CXC3R1^hi^ counterparts (Figure 1D; Figure S2F).

### CX3CR1^int^ CD8^+^ T Cells Are Induced by a Non-replicating Adenovirus Vector

**Figure 2:**
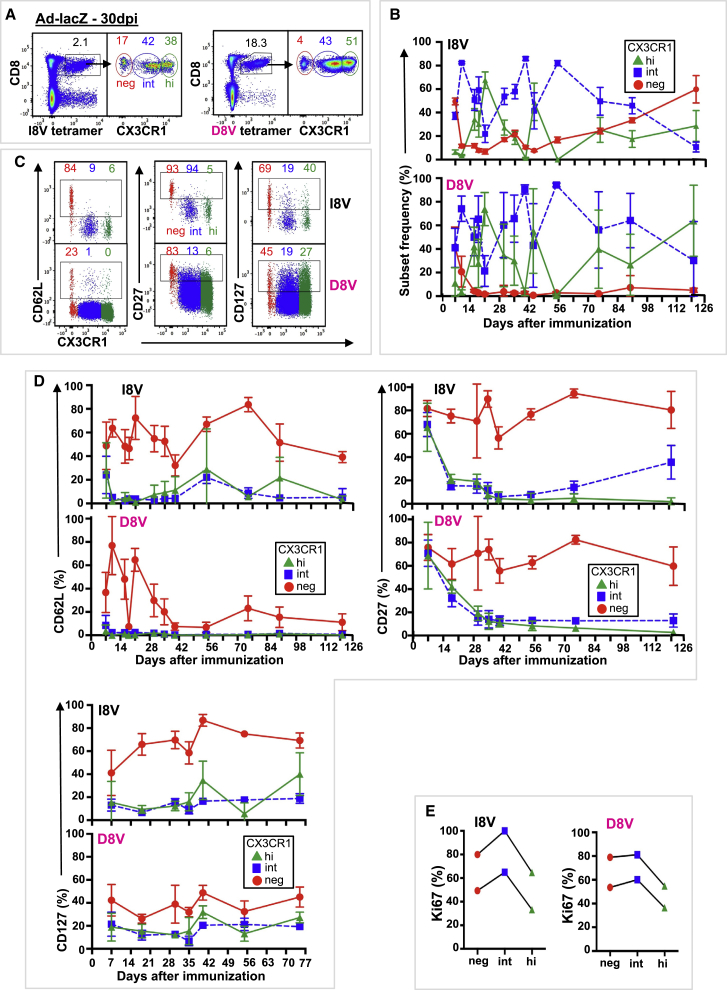
Immunization with Replication-Deficient Adenovirus Encoding MCMV Epitopes Induces Three Distinct Populations Based on CX3CR1 Expression in Conventional and Inflationary Memory CD8^+^ T Cells *Cx3cr1*^*+/gfp+*^ mice were immunized i.v. with 2 × 10^9^ PFU of a recombinant replication-deficient HuAd5 vector expressing lacZ (Ad-lacZ), and blood was serially sampled. Conventional memory responses (black label) were assessed by I8V-tetramer staining, and inflationary memory responses (pink label) were assessed D8V-tetramer staining. Coloring is consistent for all panels. (A) Composite FACS plots (n = 4) of I8V- and D8V-tetramer staining (left) and CX3CR1 subsets of tetramer^+^ CD8^+^ T cells (right) 30 dpi. Mean live tetramer^+^ CD8^+^ T cells and mean CX3CR1 subsets are indicated (n = 4). (B) Mean (±SD) CX3CR1 subsets of I8V- and D8V-tetramer^+^ CD8^+^ T cells. (C and D) Expression of surface markers CD62L, CD27, and CD127 by CX3CR1 subset on I8V- and D8V-tetramer^+^ CD8^+^ T cells. (C) Composite FACS plots (n = 5) (L to R) of CD62L, CD27, and CD127 expression by CX3CR1 subset in I8V- and D8V-tetramer^+^ CD8^+^ T cells 75 dpi, showing mean expression for each CX3CR1 subset (n = 5). (D) Mean (±SD) CX3CR1 subsets expressing CD62L (top left), CD27 (top right), and CD127 (bottom left) in I8V- and D8V-tetramer^+^ CD8^+^ T cells (n = 3–13). (E) Ki67 expression by CX3CR1 subset in I8V- and D8V-tetramer^+^ CD8^+^ T cells isolated from spleen (n = 2) 44 dpi. Data are compiled from 2–19 mice from 2–4 independent time course experiments. See also Figures S1 and S3.

To further explore the contribution of viral replication and/or antigen persistence to the distribution and phenotype of CX3CR1 Tmem subsets, we examined the qualities of vaccine-induced responses following administration of a recombinant replication-deficient Ad-lacZ (Bolinger et al., 2013). Ad-lacZ immunization induces two distinct pathways for memory: a typical contracting response to a one epitope (I8V) and an inflating response to second epitope (D8V), which is analogous to conventional (M45) and inflating (M38) responses to MCMV infection (Bolinger et al., 2013). i.v. immunization of *Cx3cr1*^*+/gfp+*^ and WT C57BL/6 mice with 2 × 10^9^ PFU Ad-lacZ resulted in two CD8^+^ Tmem responses, the conventional (contracting, I8V-tetramer^+^) and the expanded (inflationary, D8V-tetramer^+^) CD8^+^ T cell response in blood (Figure 2A; Figures S1 and S3A). CX3CR1 expression levels distinguished three CD8^+^ Tmem subsets (neg, int, and hi) (Figure 2A; Figure S3A), as with MCMV infection. Similar to MCMV infection, conventional I8V-tetramer^+^ cells maintained increasing frequencies of CX3CR1^neg^ cells, and inflating D8V-tetramer^+^ cells were nearly all CX3CR1 positive (int and hi) (Figures 2A and 2B; Figure S3B), consistent with previous reports (Bolinger et al., 2015). In the early memory phase, more conventional I8V-tetramer^+^ cells were CX3CR1 positive compared to M45-tetramer cells, which may reflect continued presentation of I8V antigen in the Ad-lacZ model. Similar to MCMV infection, Ad-lacZ-derived conventional and inflating Tmem populations contained significant frequencies of CX3CR1^int^ cells (ranges 22%–86% and 21%–94%, respectively) during the early memory phase (21–89 dpi) (Figure 2B), which had a TEM phenotype (CD62L^−^, CD27^−^, CD127^−^) (Figures 2C and 2D). During the late memory phase (>90 dpi), CX3CR1^neg^ cells dominated conventional I8V-tetramer^+^ responses (Figure 2B) and resembled TCM (CD62L^+^, CD27^+^, CD127^+^), while late inflationary D8V-tetramer^+^ T cells were mostly CX3CR1^hi^ and resembled TEM (CD62L^−^, CD27^−^, CD127^−^) (Figures 2C and 2D). As reported previously (Gerlach et al., 2016), CX3CR1^int^ cells divided more intensively than CX3CR1^neg^ and CX3CR1^hi^ cells, as measured by Ki67 staining, in both conventional and inflating memory populations (Figure 2E). A similar phenotype of CX3CR1^int^ cells in Ad-lacZ-derived conventional and inflating populations was observed in C57BL/6 WT mice (Figures S3A–S3D).

### CD8^+^ T Cells in Tissues Are CX3CR1^hi^ in the Setting of Persistent Antigen

**Figure 3:**
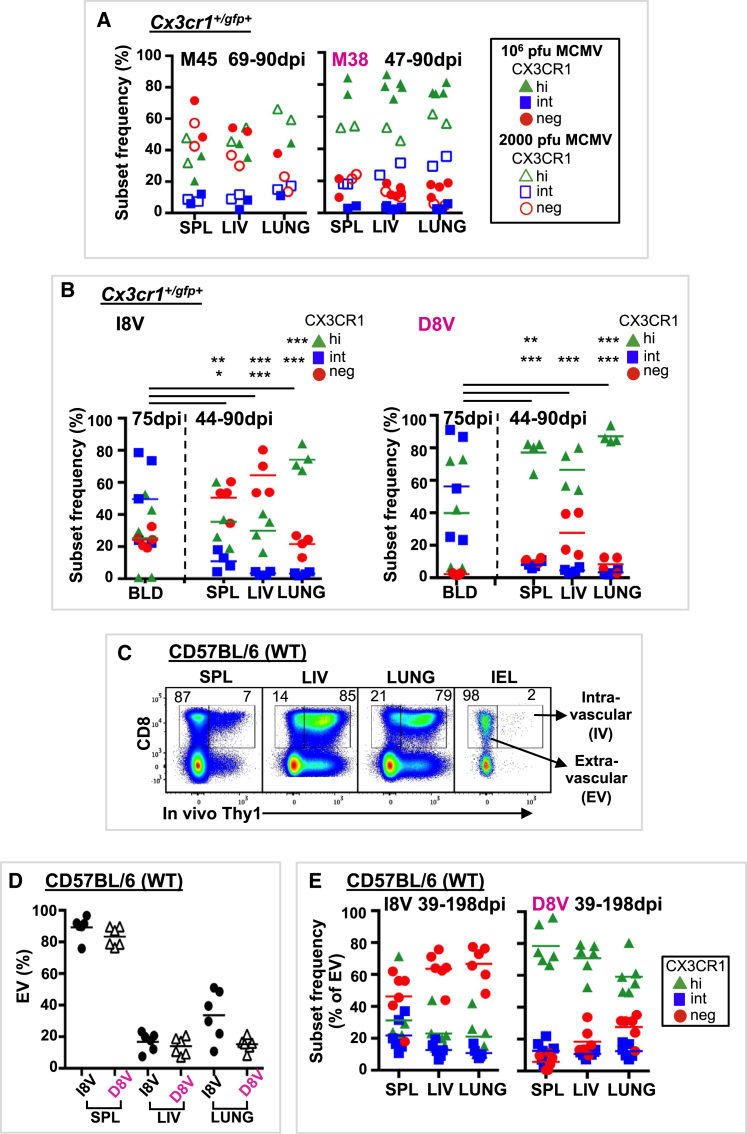
Inflationary Memory T Cells in Tissues Express High Levels of CX3CR1 following MCMV Infection or Ad-lacZ Immunization CX3CR1 subset distribution of conventional and inflationary memory CD8^+^ T cells responses was assessed in blood (BLD), spleen (SPL), liver (LIV), and lung following MCMV or Ad-lacZ infection or immunization. (A) *Cx3cr1*^*+/gfp+*^ mice were infected i.v. with 10^6^ PFU MCMV (solid symbols) or 2,000 PFU MCMV (open symbols). CX3CR1 subset frequencies for M45- and M38-tetramer^+^ CD8^+^ T cells are shown. (B) *Cx3cr1*^*+/gfp+*^ mice were immunized i.v. with 2 × 10^9^ PFU Ad-lacZ. CX3CR1 subset frequencies and means for I8V- and D8V-tetramer^+^ CD8^+^ T cells are shown. (C–E) Live lymphocytes from SPL, LIV, lung, and gut intraepithelium (IEL) labeled by (intravascular, IV) or protected from (extravascular, EV) i.v. anti-Thy1 antibodies in C57BL/6 (WT) mice 39 or 198 dpi with Ad-lacZ (n = 6 for SPL, LIV, and lung; n = 1 for IEL). (C) Representative FACS plots showing mean EV (right number) and IV (left number) CD8^+^ T cells. (D) Individual and mean EV I8V- and D8V-tetramer^+^ CD8^+^ T cells. (E) CX3CR1 subset frequencies and means of EV I8V- and D8V-tetramer^+^ CD8^+^ T cells. Statistically significant differences (SSDs) were determined by t test and corrected for multiple comparisons (Holm-Sidak). ^∗^p = 0.05 to 0.011, ^∗∗^p = 0.01 to 0. 001, ^∗∗∗^p < 0.001. Data are compiled from 3–4 independent experiments. See also Figures S2 and S3.

We next examined CX3CR1 expression in MCMV and Ad-lacZ Tmem responses in tissues (spleen, liver, and lung) of *Cx3cr1*^*+/gfp+*^ and C57BL/6 WT mice (Figure 3; Figures S2G, S2H, S3E, and S3F). The spleen, liver, and lungs are sites of viral amplification in acute MCMV infection and contain higher viral titers than the blood (Vliegen et al., 2003). We hypothesized that CX3CR1^hi^ cells would be more prevalent at these tissue sites with high levels of viral replication. CX3CR1 subset distribution in spleen, liver, and lung was distinct from that in blood at similar time points, with conventional tissue Tmem cells containing a mix of CX3CR1^neg^ and CX3CR1^hi^ cells and inflating tissue Tmem cells largely consisting of CX3CR1^hi^ cells (Figures 1B and 3A; Figure S2G). CX3CR1^int^ cells were largely absent from tissue Tmem populations; however, infection with low-dose MCMV resulted in some CX3CR1^int^ cells in the inflating population (Figure 3A).

As in MCMV infection, following Ad-lacZ vaccination, CX3CR1^int^ conventional and inflating Tmem cells were largely absent from tissues yet were present in the blood at similar time points (Figure 3B). Using *in vivo* antibody labeling to identify extravascular (i.e., unlabeled by i.v. antibody) Tmem cells (Figure 3C), conventional tissue Tmem cells were mostly CX3CR1^neg^, as reported previously (Gerlach et al., 2016), and inflating tissue Tmem cells were mostly CX3CR1^hi^ (Figures 3D and 3E). Most I8V- and D8V-tetramer^+^ cells in the liver were accessible to the circulation (i.v.) (Figures 3C and 3D) yet had high expression (82%–98%, n = 3) of the intracellular adhesion molecule 1 (ICAM-1) ligand leukocyte function-associated antigen 1 (LFA-1) (Figure S3F), regardless of CX3CR1 subset. LFA-1 expression has been shown to allow liver-resident Tmem to patrol and remain in hepatic sinusoids (McNamara et al., 2017). As has been reported (Smith et al., 2015), lung inflationary Tmem cells (M38- and D8V-tetramer^+^) did not express the tissue-resident markers CD69 and CD103 (Figure S2H). Ad-lacZ DNA and RNA can be isolated from spleen, liver, and lung at day 100 post-immunization (Bolinger et al., 2013), suggesting that vaccine antigen may be present in tissues a long time after immunization. Altogether, the similarities of CX3CR1 subset distribution and phenotype between persistent MCMV infection and immunization with a non-replicative adenoviral vector support the role of antigen persistence rather than viral replication in determining the expression and phenotype of CX3CR1 subsets on Tmem cells.

### CX3CR1 Is Required for Optimal Inflating T Cell Memory Responses following Ad-lacZ Immunization

**Figure 4:**
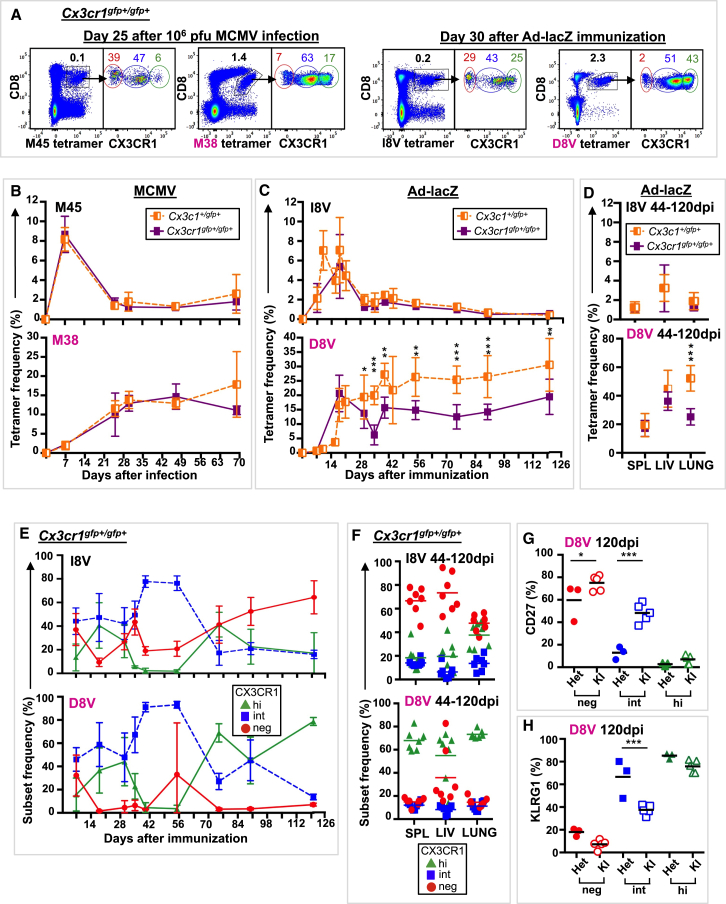
CX3CR1 Is Required for Optimal Inflating Responses following Ad-lacZ Immunization *Cx3cr1*^*gfp+/gfp+*^ mice were infected i.v. with 10^6^ PFU MCMV or 2 × 10^9^ PFU Ad-lacZ, and blood was serially sampled. (A) Composite FACS plots of M45- and M38-tetramer staining (left, n = 4) and I8V- and D8V-tetramer staining (right, n = 6) of live lymphocytes and CX3CR1 subsets of tetramer^+^ CD8^+^ T cells. Mean tetramer^+^ CD8^+^ T cells and mean CX3CR1 subsets are indicated. (B) Mean (±SD) M45- and M38-tetramer^+^ CD8^+^ T cells in blood from *Cx3cr1*^*+/gfp+*^ mice (orange half-filled square, n = 2–3) and *Cx3cr1*^*gfp+/gfp+*^ mice (purple-filled square, n = 3–4). (C) Mean (±SD) I8V- and D8V-tetramer^+^ CD8^+^ T cells in blood from *Cx3cr1*^*+/gfp+*^ mice (n = 3–19) and *Cx3cr1*^*gfp+/gfp+*^ mice (n = 3–15). (D) Mean (±SD) I8V- and D8V-tetramer^+^ CD8^+^ T cells in spleen (SPL), liver (LIV), and lung from *Cx3cr1*^*gfp+/gfp+*^ mice (n = 4–5) and *Cx3cr1*^*gfp+/gfp+*^ mice (n = 7). (E) Mean (±SD) CX3CR1 subsets of I8V- and D8V-tetramer^+^ CD8^+^ T cells in blood of *Cx3cr1*^*gfp+/gfp+*^ mice (n = 3–15). (F) CX3CR1 subset frequencies and mean of I8V- and D8V-tetramer^+^ CD8^+^ T cells in spleen (SPL), liver (LIV), and lung from *Cx3cr1*^*gfp+/gfp+*^ mice. (G and H) Expression of CD27 (G) and KLRG1 (H) by CX3CR1 subset in D8V-tetramer^+^ CD8^+^ T cells in *Cx3cr1*^*+/gfp+*^ (HET, n = 3) and *Cx3cr1*^*gfp+/gfp+*^ (KI, n = 5) mice 120 dpi. Shown are individual values and mean. SSDs were determined by t test and corrected for multiple comparisons (Holm-Sidak). ^∗^p = 0.05 to 0.011, ^∗∗^p = 0.01 to 0. 001, ^∗∗∗^p < 0.001. Data are compiled from 3–5 independent experiments. See also Figure S4.

The previous study has indicated that CX3CR1 is not required for antigen recognition or effector differentiation following acute viral infection (Gerlach et al., 2016). We next explored whether CX3CR1 is required for Tmem development in persistent MCMV infection and/or immunization with Ad-lacZ. We used *Cx3cr1*^*gfp+/gfp+*^ mice in which GFP was knocked in to both *Cx3cr1* loci; therefore, functional CX3CR1 protein is not expressed. Mice were infected with high-dose MCMV, low-dose MCMV, or Ad-lacZ, as described previously. *Cx3cr1*^*gfp+/gfp+*^ mice generated robust conventional and inflating Tmem responses in blood (Figures 4A and 4B) and tissues (Figure S4B) following high- and low-dose MCMV infection with similar CX3CR1 subset distribution to that of *Cx3cr1*^*+/gfp+*^ mice (Figures S4A and S4C). In contrast, CX3CR1 was required for optimal magnitude of inflating (D8V^+^) Tmem responses, but not conventional (I8V^+^) Tmem responses, following Ad-lacZ immunization in blood (Figure 4C) and tissues (Figure 4D). Further characterization of D8V^+^ cells from *Cx3cr1*^*gfp+/gfp+*^ mice revealed a CX3CR1 subset distribution similar to that for *Cx3cr1*^*+/gfp+*^ mice in blood (Figures 2B and 4E) and tissues (Figures 3B and 4F) but increased CD27 (Figure 4G) and decreased KLRG1 (Figure 4H) expression over time in CX3CR1^int^ subsets. CX3CR1-expressing macrophages are thought to be involved in the spread of MCMV (Daley-Bauer et al., 2014); however, because differences were not observed in the conventional memory (M45- and I8V-tetramer^+^) populations, which act as an internal control (Figures 4A–4F; Figure S4), viral spread in *Cx3cr1*^*gfp+/gfp+*^ mice is likely to be preserved. No differences were observed in the number of CD8^+^ T cells in tissues between *Cx3cr1*^*+/gfp+*^ and *Cx3cr1*^*gfp+/gfp+*^ mice, indicating that T cell localization remains intact (Table S1). These results suggest that CX3CR1 is not required for memory inflation but is required for optimal responses and effector differentiation of inflating vaccine-derived Tmem cells.

### Distribution and Phenotype of Three CX3CR1 Subsets in Human CD8^+^ T Cells

**Figure 5:**
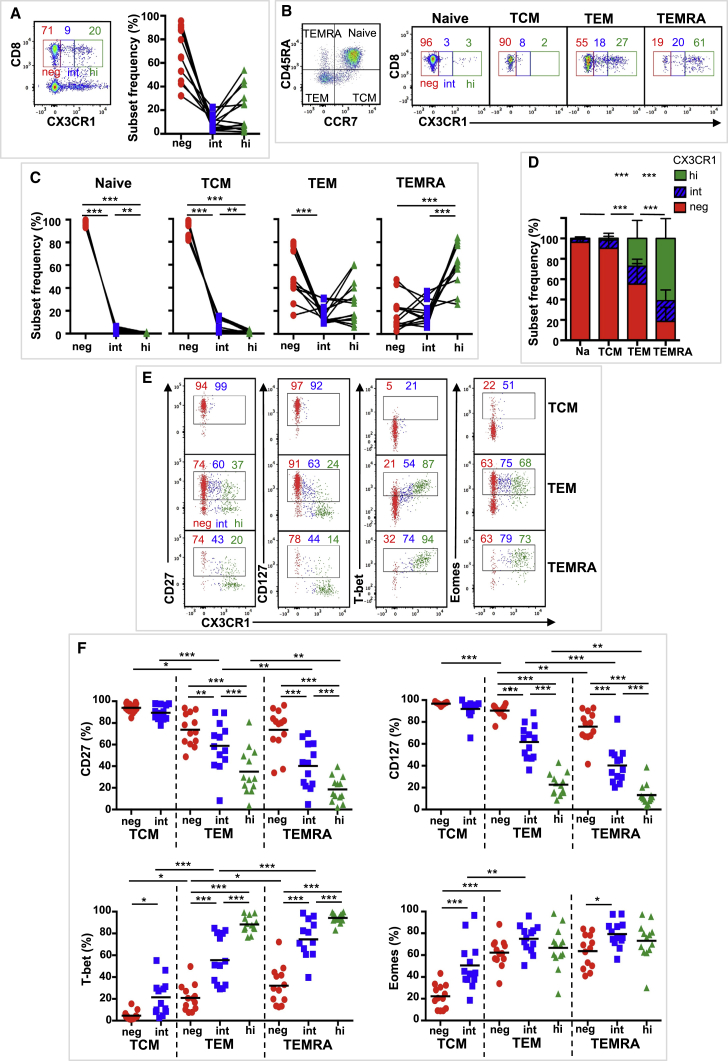
Three Distinct Human CD8^+^ T Cell Populations Based on CX3CR1 Expression CD8^+^ T cell expression of CX3CR1 was assessed in blood obtained from 13 volunteers. (A) Composite FACS plot (n = 5) of CX3CR1 expression gated on live CD3^+^ lymphocytes (left) with mean CX3CR1 subset frequency of CD8^+^CD3^+^ T cells indicated (n = 13). Individual CX3CR1 subset frequencies are shown on the right (n = 13). (B) Left: CD8^+^ T cell subsets defined as CD45RA^+^/CCR7^+^ (naive), CD45RA^−^/CCR7^+^ (central memory; TCM), CD45RA^−^/CCR7^−^ (effector memory; TEM), and CD45RA^+^/CCR7^−^ (terminal effector; TEMRA). Right: composite FACS plots (n = 5) of CX3CR1 expression in naive, TCM, TEM, and TEMRA CD8^+^ T cells. CX3CR1 subset means are indicated (n = 13). (C) Individual CX3CR1 subset frequencies in naive, TCM, TEM, and TEMRA CD8^+^ T cells (n = 13). (D) Mean (±SD) CX3CR1 subsets in naive, TCM, TEM, and TEMRA CD8^+^ T cells (n = 13). (E and F) CX3CR1 subset expression of cell surface markers (CD27 and CD127) and transcription factors (T-bet, Eomes) in CD8^+^ Tmem cells. (E) Composite FACS plots (n = 5) (L to R) of CD27, CD127, T-bet, and Eomes expression by CX3CR1 subsets on TCM, TEM, and TEMRA CD8^+^ T cells. Mean expression for each CX3CR1 subset is indicated (n = 13). (F) Individual and mean CX3CR1 subset expression (L to R) of CD27, CD127, T-bet, and Eomes in TCM, TEM, and TEMRA CD8^+^ T cells (n = 13). SSDs were determined by two-way ANOVA and corrected for multiple comparisons (Holm-Sidak). ^∗^p = 0.05 to 0.011, ^∗∗^p = 0.01 to 0. 001, ^∗∗∗^p < 0.001. Data are compiled from a single experiment. See also Figure S5.

We next addressed whether such changes in CX3CR1 expression and phenotype can be observed in human memory pools induced by CMV and adenoviral vectors. There are limited human data describing the distribution and phenotype of CX3CR1 subsets in polyclonal CD8^+^ T cells. To address this knowledge gap, we first focused on whether CX3CR1 expression can distinguish three CD8^+^ Tmem cell subsets in humans by analyzing peripheral blood mononuclear cells from 13 volunteers aged 21–54 years (Table S2). Samples were collected as part of adenoviral-vaccine studies (n = 10) or from healthy volunteers (n = 3). Polyclonal CD8^+^ T cell subset distribution and CX3CR1 expression did not differ during the vaccine study, and the end-of-study (EOS) sample was used for analysis. CX3CR1 expression levels distinguished three CX3CR1 subsets (neg, int, and hi) on bulk CD8^+^ T cells (Figure 5A; Figure S5A). Delineation of CD8^+^ T cells into subsets based on CD45RA and CCR7 expression (naive, CD45RA^+^/CCR7^+^; TCM, CD45RA^−^/CCR7^+^; TEM, CD45RA^−^/CCR7^−^; and terminal effector [TEMRA], CD45RA^+^/CCR7^−^), showed variation in the CX3CR1 population distribution within each subset (Figures 5B–5D). TEM and TEMRA cells had comparative frequencies of CX3CR1^int^ cells (17% and 19%, respectively) (Figure 5D), while CX3CR1^neg^ were more prevalent in TEM cells (i.e., CX3CR1neg > hi > int) and CX3CR1^hi^ cells were more prevalent in TEMRA cells (i.e., CX3CR1hi > neg = int). Naive and TCM cells were mostly CX3CR1^neg^, with some CX3CR1^int^ cells in the TCM subset (Figures 5B–5D). We next analyzed the phenotypic markers CD27 and CD127 and the transcription factors T-bet and Eomes (Figures 5E and 5F). Most TEM and TEMRA CX3CR1^int^ cells had features of effector memory and antigen-mediated activation (CD27^−^, CD127^−^, T-bet^+^), which were more pronounced in the TEMRA population. Overall, the phenotype of CX3CR1^int^ cells represented a mid-point between the CX3CR1^neg^ and the CX3CR1^hi^ populations, consistent with CD8^+^ T cell differentiation being a continuum (Figure 7F) (Wong et al., 2016).

### Human Adenoviral Vaccine and Persistent CMV Infection Induce CX3CR1^int^ Antigen-Specific CD8^+^ Tmem Cells

To assess how CX3CR1 subsets on Tmem cells relate to persistent infection and adenoviral-vaccine-induced responses in humans, we analyzed (1) CD8^+^ T cells primed using a chimpanzee adenovirus strategy (ChAd3-NSmut), specific for dominant HCV epitopes from NS3 (NS395, HLA-A2 restricted, and NS3103, HLA-A1 restricted) (Swadling et al., 2014), and (2) CMV-specific CD8^+^ T cells.

**Figure 6:**
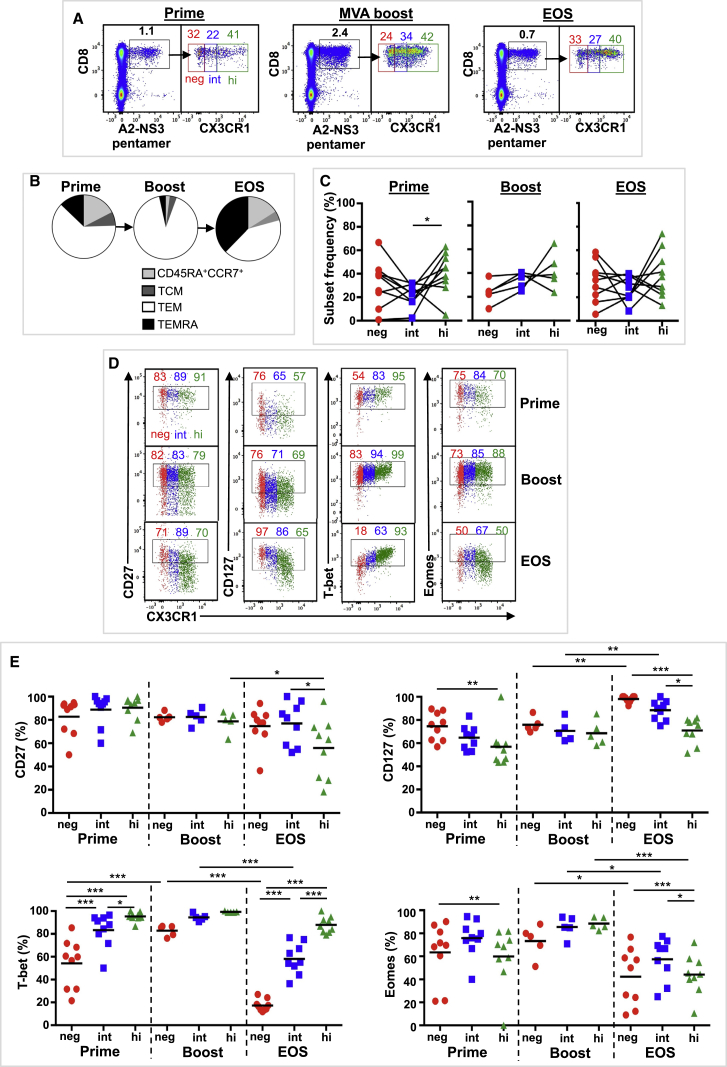
Immunization with Replicative Defective Adenoviral Vectors Induces Three Subsets Based on CX3CR1 Expression in CD8^+^ Memory T Cells in Humans CX3CR1 expression was assessed in adenoviral vaccine-derived CD8^+^ T cells in blood obtained from 9 volunteers. Blood was taken at peak response after ChAd3-NSmut prime (trial weeks [TW] 2–4, prime, n = 9), peak response after MVA-NSmut boost (TW9, boost, n = 5), and at end of study (TW16–TW32, EOS, n = 9). Vaccine-derived CD8^+^ T cells were identified by staining with an HLA-A2 pentamer containing an HCV NS3 epitope (A2-NS3 pentamer). (A) Composite FACS plots of A2-NS3 pentamer staining (left) of live CD3^+^ lymphocytes and CX3CR1 subsets of A2-NS3 pentamer^+^ CD8^+^ T cells. Mean A2-NS3 pentamer^+^ CD8^+^ T cell frequency and mean CX3CR1 subset frequency are indicated. (B) Pie chart showing mean CD8^+^ T cell subset distribution of A2-NS3 pentamer^+^ CD8^+^ T cells. (C) CX3CR1 subset frequency of A2-NS3 pentamer^+^ CD8^+^ T cells. (D and E) CX3CR1 subset expression of cell surface markers (CD27 and CD127) and transcription factors (T-bet and Eomes) in A2-NS3 pentamer^+^ CD8^+^ T cells. (D) Composite FACS plots (L to R) of CD27, CD127, T-bet, and Eomes expression on A2-NS3 pentamer^+^ CD8^+^ T cells. Mean expression for each CX3CR1 subset is indicated. (E) Individual values and mean CX3CR1 subset expression (L to R) of CD27, CD127, T-bet, and Eomes in A2-NS3 pentamer^+^ CD8^+^ T cells. SSDs were determined by one-way ANOVA (C) or two-way ANOVA (E) and corrected for multiple comparisons (Holm-Sidak). ^∗^p = 0.05 to 0.011, ^∗∗^p = 0.01 to 0.001, ^∗∗∗^p < 0.001. Data are compiled from a single time course experiment. See also Figure S5.

CX3CR1 expression was assessed in adenoviral vaccine-induced CD8^+^ T cells in blood obtained from 9 volunteers, aged 21–54 years (Table S2). All volunteers received a priming regimen of ChAd3-NSmut (5 × 10^8^–2.5 × 10^10^ viral particles) encoding the NS3, NS4, NS5A, and NS5B proteins of HCV genotype 1b (Figure S5B) (Swadling et al., 2014). A subset of 5 volunteers also received a boost with a modified vaccinia Ankara (MVA)-NSmut (2 × 10^7^–2 × 10^8^ PFU) vector encoding the same HCV genotype 1b proteins 8 weeks later (Figure S5; Table S2). Blood samples were taken at peak response after ChAd3-NSmut prime (trial weeks [TW] 2–4, prime), peak response after MVA-NSmut boost (TW9, boost), and at end of study (EOS) (TW16–TW32). Peak vaccine responses were determined by ELISpot as described previously (Swadling et al., 2014). Vaccine-derived CD8^+^ T cells were assessed by staining with HLA-A2 pentamers containing an NS3 epitope (A2-NS3 pentamer) (Table S3). All volunteers had detectable A2-NS3 pentamer^+^ T cells (defined as ≥0.10% CD8^+^ T cells) after priming, which increased in frequency following MVA boost and contracted by EOS (Figure 6A), as reported previously (Swadling et al., 2014). No differences in NS3 pentamer^+^ CX3CR1 subsets were observed at EOS in those that received MVA boost and those that did not (Figure S5C), and both groups were combined for the EOS analysis. NS3 pentamer^+^ T cells were predominantly TEM cells following prime and boost immunization, with TEMRA cells becoming more prevalent by EOS (Figure 6B) (Swadling et al., 2014). CX3CR1 expression and phenotype were assessed in total NS3 pentamer^+^ CD8^+^ T cells because of the low number of NS3 pentamer^+^ T cells analyzed per memory subset. CX3CR1 expression levels distinguished three NS3 pentamer^+^ T cell CD8^+^ T subsets (Figure 6A), resembling the CX3CR1 subset distribution pattern observed in polyclonal CD8^+^ TEM and TEMRA cells (Figure 5C). At all time points, CX3CR1^int^ NS3 pentamer^+^ cells were detected at stable mean frequency (range 22%–37%). CX3CR1^hi^ NS3 pentamer^+^ cells were more prevalent than CX3CR1^int^ NS3 pentamer^+^ cells following prime, and no differences in CX3CR1 subset distribution were observed at other time points. Analysis of phenotypic markers CD27 and CD127 and transcription factors T-bet and Eomes (Figures 6D and 6E) shows a TEM phenotype (T-bet^+^) in all CX3CR1 subsets after boost and, to a lesser extent, after prime. By EOS, the phenotype of NS3 pentamer^+^ CX3CR1 subsets resembled polyclonal TEM and TEMRA populations. These results indicate that human adenovector vaccination can induce and sustain all three CX3CR1 subsets with a similar phenotype to polyclonal CD8^+^ TEM cells. A persistent mixed memory population of TEM, TCM, and TEMRA cells is indicative of a functional memory response induced by adenovectors and is characterized by increasing CD127 expression, T-bet retention, and reducing Eomes expression (Swadling et al., 2014).

**Figure 7:**
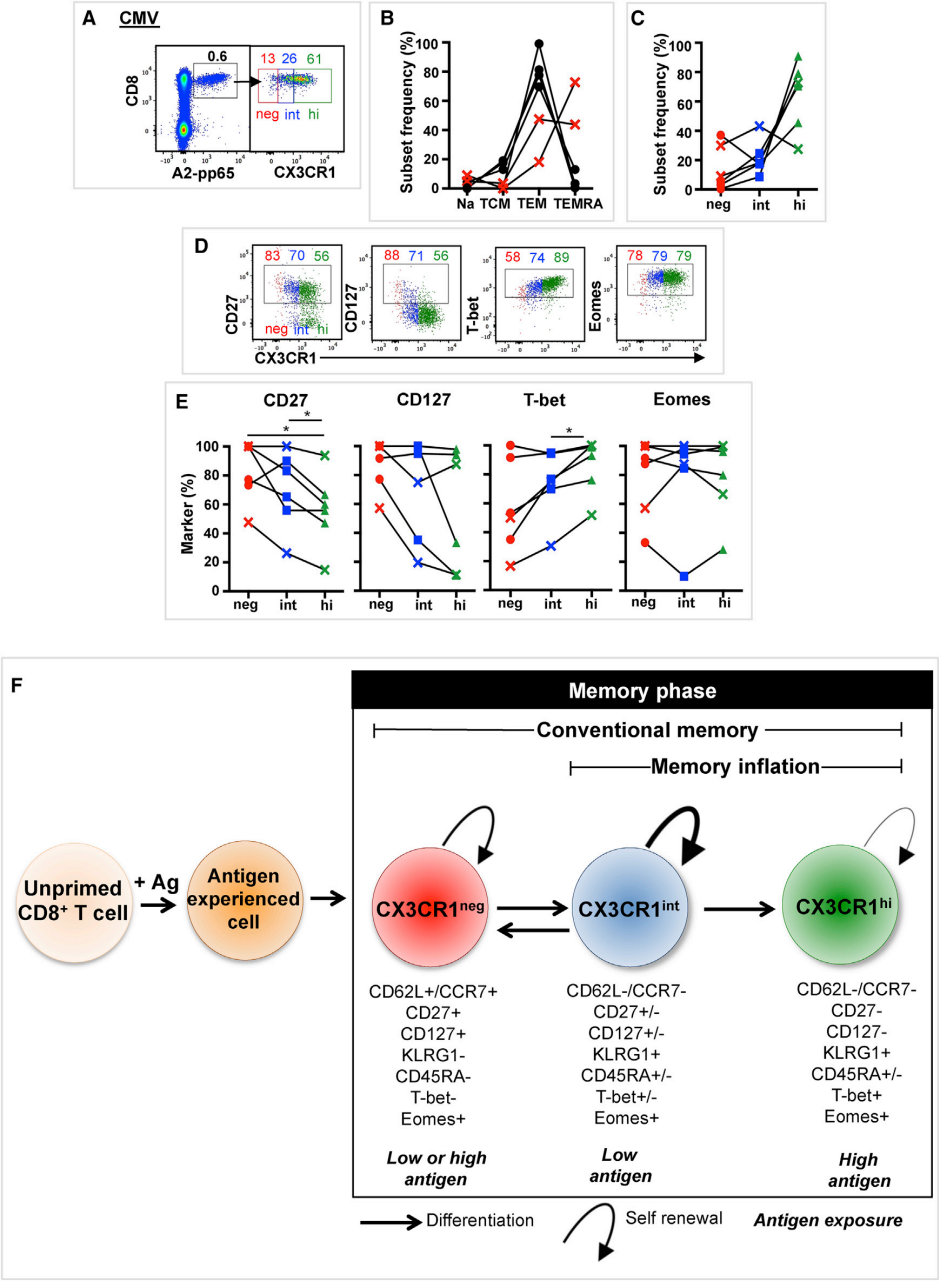
Natural Infection with Human CMV Induces Three CD8^+^ T Cell Subsets Based on CX3CR1 Expression CX3CR1 expression was assessed in CMV-specific CD8^+^ T cells in blood obtained from 6 volunteers. CMV-specific CD8^+^ T cells were identified by tetramer staining (Table S3). (A) Composite FACS plots (n = 3) of A2-pp65 tetramer staining (left) of live CD3^+^ lymphocytes and CX3CR1 subsets (right) of A2-pp65 tetramer ^+^ CD8^+^ T cells. Mean CMV tetramer^+^ CD8^+^ T cell frequency and mean CX3CR1 subset frequency are indicated (n = 6). (B) Individual CD8^+^ T cell subset distributions of CMV tetramer^+^ CD8^+^ T cells (n = 6). Volunteers with TEMRA-dominant CMV tetramer^+^ populations are indicated by a red cross. (C) CX3CR1 subset frequency distributions of CMV tetramer^+^ CD8^+^ T cells. (D) Composite FACS plots (n = 3) (L to R) of CD27, CD127, T-bet, and Eomes expression on CMV tetramer^+^ CX3CR1 subsets. Mean percentage of marker expression for each CX3CR1 subset is indicated (n = 6). (E) CMV tetramer^+^ CX3CR1 subsets expressing (L to R) CD27, CD127, T-bet, and Eomes. A cross indicates a TEMRA-dominant CMV tetramer^+^ population. SSDs were determined by one-way ANOVA and corrected for multiple comparisons (Holm-Sidak). ^∗^p = 0.05 to 0.011, ^∗∗^p = 0.01 to 0. 001, ^∗∗∗^p < 0.001. Data are compiled from a single experiment. (F) Hypothetical model for linear CD8^+^ T cell differentiation. CX3CR1 subsets are represented, as well as their phenotypic and functional characteristics and antigen exposure requirements. +, high expression; −, no expression; +/−, intermediate expression.

We next assessed CX3CR1 expression in CMV-specific CD8^+^ T cells in blood obtained from 6 volunteers, aged 21–54 years (Table S2). Three volunteers also received the adenovirus vaccine, and their EOS sample was used for the CMV analysis (no difference was observed in CMV tetramer^+^ T cells and CX3CR1 expression at any trial time point) (data not shown). CMV-specific T cells were identified by staining with HLA-matched tetramers or pentamers containing immunodominant CMV epitopes (Table S3). CMV tetramer^+^ CD8 T cells were mostly CX3CR1^int^ (26%) or CX3CR1^hi^ (61%) (Figures 7A–7C). Analysis of phenotypic markers CD27 and CD127 and transcription factors T-bet and Eomes (Figures 7D and 7E) revealed heterogenous expression patterns that were not related to magnitude of CMV tetramer^+^ response or dominance of TEM or TEMRA response. These results suggest that CX3CR1 expression and phenotype on CMV-specific T cells form a dynamic process responsive to an individuals’ CMV latency and reactivation events.

## Discussion

The induction and maintenance of robust CD8^+^ T cell memory are important for the control of persistent infection and critical to the success of vaccines against infections and cancers. The identification of CX3CR1^int^ TPM cells, a Tmem subset with self-renewal, proliferative, memory pool maintenance, and tissue-surveying properties, prompted us to hypothesize that CX3CR1^int^ TPM cells underlie the ability of CMV and adenoviral vectors to induce and sustain large inflationary CD8^+^ Tmem responses. By profiling CX3CR1 expression on MCMV- and adenovirus vaccine-specific memory CD8^+^ T cells, we identified high levels of inflationary CX3R1^int^ cells in the early memory phase compared to conventional CD8^+^ T cell memory. The CX3CR1^int^ memory pool showed a TEM phenotype characteristic of inflationary pools but showed delayed differentiation. In humans, administration of an adenovirus-vectored vaccine for HCV induced and maintained CX3CR1^int^ CD8^+^ T cell populations associated with a memory phenotype similar to that in mice and in natural HCMV infection.

Using *Cx3cr1*^*+/gfp+*^ reporter mice, Gerlach et al. (2016) reported that approximately 15% of circulating conventional memory cells were CX3CR1^int^ following acute viral infection (LCMV and vesicular stomatitis virus [VSV]) and that CX3CR1^int^ displayed some features closer to CX3CR1 ^neg^ TCM cells (similar proliferation, CD27^+^, and CD127^+^) while other functional abilities (IL-2 production, cytotoxicity, and lymph node [LN] homing) were intermediate between CX3CR1^hi^ TEM and CX3CR1^neg^ TCM cells. Also using *Cx3cr1*^*+/gfp+*^ reporter mice, we found that even larger circulating populations of CX3CR1^int^ cells are induced and sustained in early inflating memory populations following MCMV infection and Ad-lacZ immunization (range 21%–94%) and that inflating CX3CR1^int^ cells have an effector phenotype similar to that of their CX3CR1^hi^ counterparts. By comparison, CX3CR1^int^ conventional memory populations following MCMV infection and Ad-lacZ immunization (acting as internal controls) shared similar features to the CX3CR1^int^ conventional memory cells described by Gerlach et al. (2016). Previous data have indicated that inflationary memory cells are derived from the TCM pool (Smith et al., 2014). Our data suggest that CX3CR1^int^ cells, either derived from CX3CR1^neg^ TCM or self-renewing, contribute to stably maintaining the expanding size and effector phenotype of the inflating memory population (Figure 7F).

The initial sustained inflating CX3CR1^int^ response observed in our models is likely linked to low-level antigen persistence, possibly by presentation on non-professional antigen-presenting cells (APCs). The current model for memory inflation involves repetitive antigen presentation occurring on non-classical APCs, potentially in the vascular endothelium or a non-hematopoietic lymph node cell (Smith et al., 2014, Torti et al., 2011). To support the role of low-level antigen persistence in inducing inflating CX3CR1^int^ cells, we found that (1) by reducing the infecting dose of MCMV, circulating inflating CX3CR1^int^ cells became even more frequent; (2) in tissues where viral replication is amplified—and to lesser extent, when a reduced infecting dose was used—CX3CR1^hi^ inflating cells remained dominant; and (3) inflating CX3CR1^int^ Tmem cells have a TEM phenotype similar to their CX3CR1^hi^ inflating counterparts, suggesting ongoing antigen exposure. Preservation of these characteristics in the inflating memory pool induced by a non-replicating Ad-lacZ vaccine supports the notion that persistent antigen exposure, rather than viral replication, promotes CX3CR1^int^ expression and phenotype. Conversely, the waning of the CX3CR1^int^ population at late time points may be due to a loss of antigen exposure.

Gerlach et al. (2016) describes CX3CR1^int^ TPM cells as the dominant Tmem subset surveying tissues, rather than CX3CR1^hi^ TEM cells, in a revision of a long-held paradigm. In our mouse models where prolonged antigen exposure in tissues is a key feature, CX3CR1^int^ cells were largely absent from tissues, suggesting that inflating CX3CR1^hi^ cells continue to play a role in local tissue responses in settings of sustained antigen exposure.

Adenovirus-vectored HCV vaccination strongly induced and maintained CX3CR1^int^ CD8^+^ T cell populations (22%–34% of responses) with TEM characteristics, which overlapped with CX3CR1^int^ cells in natural HCMV infection and inflated populations in MCMV and adenovirus-vector models. Furthermore, CMV- and adenovirus-vector-induced CX3CR1^int^ Tmem cells displayed the same TEM characteristics as those typically associated with viral control (Swadling et al., 2014). Tmem cells expressing CX3CR1 are mostly absent from patients with chronic HCV infection, and the few present have markers of cytotoxicity (Böttcher et al., 2015). In combination with the previously reported qualities of self-renewal, maintenance of memory pools, and tissue surveillance, CX3CR1^int^ cells may improve efficacy and endurance of CD8^+^ T cells vaccine responses.

CX3CR1 appears to be an important marker for distinct T cell memory subsets in mouse and human. We also explored the requirement for CX3CR1 in the generation of memory inflation in both MCMV and adenoviral models using *Cx3cr1*^*gfp+/gfp+*^ mice. We showed that CX3CR1 is not required for memory inflation or conventional memory induction in the MCMV model. However, in the Ad-lacZ model, some blunting of memory cell frequencies and differentiation were seen following vaccination. Thus, CX3CR1 does not overall play a non-redundant role in memory formation, although the signal from the adenovirus-vector experiments suggests it may play a role in maintenance of inflated populations. This difference between the two models likely relates to the continuous replication of MCMV and the additional inflammatory responses. For example, MCMV-driven memory inflation is relatively unaffected by lack of CD4^+^ T cell help, whereas adenovirus-vector-induced memory is affected (Provine et al., 2014, Snyder et al., 2009).

Although the human and mouse data are broadly consistent, we observed some differences. We distinguished three CX3CR1 subsets (neg, int, and hi) on human polyclonal CD8^+^ Tmem cells. In contrast to mice, absence of CX3CR1 expression did not define human TCM cells; 55% of TEM and 19% of TEMRA cells were CX3CR1^neg^. CX3CR1^int^ cells represented approximately 20% of both TEM and TEMRA subsets and displayed features that were intermediate between CX3CR1^hi^ and CX3CR1^neg^ cells within that CD8^+^ subset (i.e., differentiation, CD127 [IL-7R] expression, and effector function). Overall, the transition from CX3CR1^neg^ to CX3CR1^hi^ via CX3CR1^int^ maps onto other T cell differentiation markers in both cases, although the relationship is not exact and cells may transition from one state to another via distinct routes, leading to the continuum of differentiation observed using high-content cytometric approaches (Swadling et al., 2014, Wong et al., 2016).

Overall, we found that CX3CR1^int^ memory cells form a substantial component of the memory pool in response to persistent viruses and vaccines in both mouse and human. CX3R1^int^ inflating memory populations observed in the early memory phase in mice are likely linked to low-level antigen presentation on non-classical APCs, a feature that could be harnessed for promoting CX3CR1^int^ responses in humans. By identifying CX3CR1^int^ cells following adenovirus-vector vaccination, we demonstrate the feasibility of targeting and tracking these cells in CD8^+^ T cell vaccine strategies. The long-term self-renewal properties of CX3CR1^int^ TPM cells may be of relevance to the longevity, maintained effector functions, and protective capacity of these pools.

## Experimental Procedures

### Animals

Mouse experiments were performed according to UK Home Office regulations (PPL 30/3293 and 30/2744) and after approval by the University of Oxford ethical review board. Mice were kept under conventional conditions in individually ventilated cages and fed normal chow diet. C57BL/6 (Envigo, Oxford, UK) and *Cx3cr1*^*gfp+/gfp+*^ transgenic knockin mice (provided by Prof. Fiona Powrie, University of Oxford) were used. *Cx3cr1*^*+/gfp+*^ mice were derived by breeding *Cx3cr1*^*gfp+/gfp+*^ mice with outbred C57BL/6 mice (Envigo, Oxford, UK). Male and female mice were infected or immunized when aged 6–8 weeks.

### MCMV and Adenoviral Vector

The MCMV strain (Strain Smith; ATCC: VR194) was provided by Prof. U.H. Koszinoswki (Max von Pettenkofer Institute). MCMV was propagated and titrated on NIH 3T3 cells (European Collection of Authenticated Cell Cultures [ECACC], Porton Down, UK), stored at −80°C, and 2,000 or 1 × 10^6^ PFU i.v. injected. Replication-deficient recombinant adenovirus expressed the β-galactosidase (β-gal) protein under the control of the HCMV promoter (Ad-lacZ) (Bolinger et al., 2013). Ad-lacZ was propagated as previously described (Bolinger et al., 2013, Lee et al., 2017).

### Human Vaccination Protocols

The Ad6, ChAd3, and MVA vectors encoding the NS3-5B region of HCV genotype 1B (NSmut) based on sequence accession number M58335, and vaccination schedules have been described previously (Barnes et al., 2012, Swadling et al., 2014) (NCT: NCT01070407 and NCT01296451). All volunteers gave written informed consent before enrollment, and the studies were conducted according to the principles of the Declaration of Helsinki and in accordance with good clinical practice.

### Detection and Analysis of Virus and Vaccine-Specific T Cells

Tetramers and pentamers are shown in Table S3. Murine MHC class I monomers complexed with M38 (H-2Kb), M45 (H-2Db), β-gal D8V (H-2Kb), and I8V (H-2Kb) and some human MHC class I monomers (A2-pp65, A1-pp50, and B7-pp65) were tetramerized by addition of streptavidin-phycoerythrin (PE) (BD Biosciences) or streptavidin-APC (Invitrogen). Peptide was obtained from Proimmune (Oxford, UK). Aliquots of 100 μL of whole blood (mouse) or thawed 1 × 10^6^ cells (human) were stained using a 50 μL solution of tetramers and CX3CR1 antibody at 37°C for 20 min followed by monoclonal antibody (mAb) staining. Human pentamer (A2-NS3 and A2-pp65; Proimmune, Oxford, UK) staining has been described previously (Swadling et al., 2014).

### Flow Cytometry Analysis and *In Vivo* Antibody Labeling

Fluorochrome-conjugated antibodies are shown in Table S4. For *in vivo* antibody labeling, mice were injected i.v. with 3 μg fluorochrome-conjugated anti-Thy1.2 (CD90.2) 10 min before tissue harvest, and lungs were perfused with 15 mL PBS. For intranuclear staining, surface-stained cells were fixed, washed, resuspended in permeabilization buffer (eBioscience FoxP3 staining kit), and stained with anti-T-bet and Eomes antibodies. Cells were analyzed by flow cytometry using a BD LSR II flow cytometer and FlowJo (Tree Star) and gated on viable leukocytes using live/dead fixable near-infra-red (IR) (Invitrogen). Composite flow cytometer plots were generated by randomly selecting equal numbers of each cell population of interest from each subject or from all cells from each subject for populations with low cell number (i.e., pentamer^+^ cells).

### Statistical Analysis

Statistical analysis was performed using GraphPad Prism (GraphPad, La Jolla, CA). The p values for comparison of means were determined by two-tailed t test and one-way and two-way ANOVA and were corrected using Holm-Sidak for multiple comparisons.

## Supplemental Information

Document S1. Figures S1–S5 and Tables S1–S4

Document S2. Article plus Supplemental Information
