# Supplementary material for: Induction and Maintenance of CX3CR1-Intermediate Peripheral Memory CD8+ T Cells by Persistent Viruses and Vaccines
Source: Cell Rep. 2018 Apr 17;23(3):768–82. doi: 10.1016/j.celrep.2018.03.074 (PMC5917822; doi:10.1016/j.celrep.2018.03.074)
Supplement: Document S1. Figures S1–S5 and Tables S1–S4 [file mmc1.pdf]

Cell Reports, Volume *23*

Supplemental Information

Induction and Maintenance of CX3CR1-Intermediate Peripheral Memory $CD8^+$ T Cells by Persistent Viruses and Vaccines

Claire Louse Gordon, Lian Ni Lee, Leo Swadling, Claire Hutchings, Madeleine Zinser, Andrew John Highton, Stefania Capone, Antonella Folgori, Eleanor Barnes, and Paul Klenerman

Figure S1

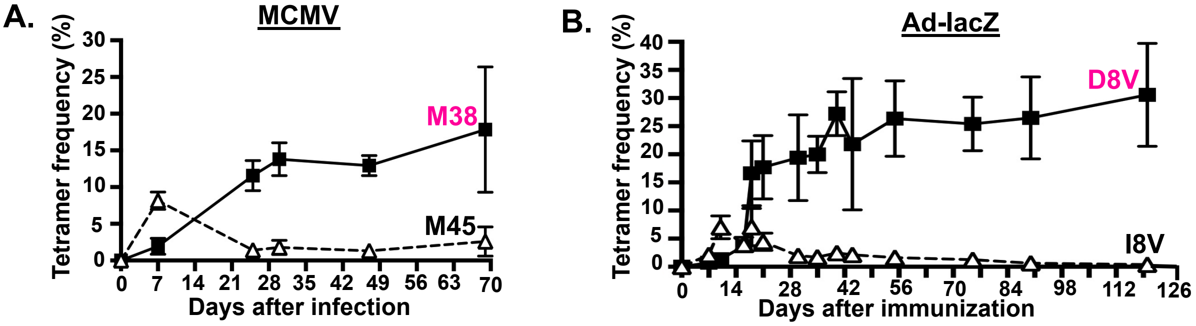

A.
MCMV
Tetramer frequency (%)
30
25
20
15
10
5
0
M38
M45
0 7 14 21 28 35 42 49 56 63 70
Days after infection
B.
Ad-lacZ
Tetramer frequency (%)
40
35
30
25
20
15
10
5
0
D8V
I8V
0 14 28 42 56 70 84 98 112 126
Days after immunization

Figure S1: MCMV infection and Ad-lacZ immunization induces conventional and inflating $CD8^+$ T cell memory responses, related to Figures 1 and 2

Cx3cr1$^{+/gfp+}$ mice were infected intravenously (iv) with 10^6 pfus MCMV or $2x10^9$ pfu Ad-lacZ and blood was serially sampled post-immunization (dpi). Conventional memory responses (black label) were assessed by M45 (MCMV)- and I8V(Ad-lacZ)- tetramer staining, and inflationary memory responses (pink label) were assessed M38(MCMV)- and D8V(Ad-lacZ)- tetramer staining (coloring is consistent for all Supplemental Figures). (A) Compiled results showing mean (±SD) tetramer frequency of M45- (open triangle, dotted line) and M38- (filled square, solid line)-tetramer$^+$ $CD8^+$ T cells (n=2-4). (B) Compiled results showing mean (±SD) I8V- (open triangle, dotted line) and D8V- (filled square, solid line) tetramer$^+$ $CD8^+$ T cells (n=3-19).

Figure S2

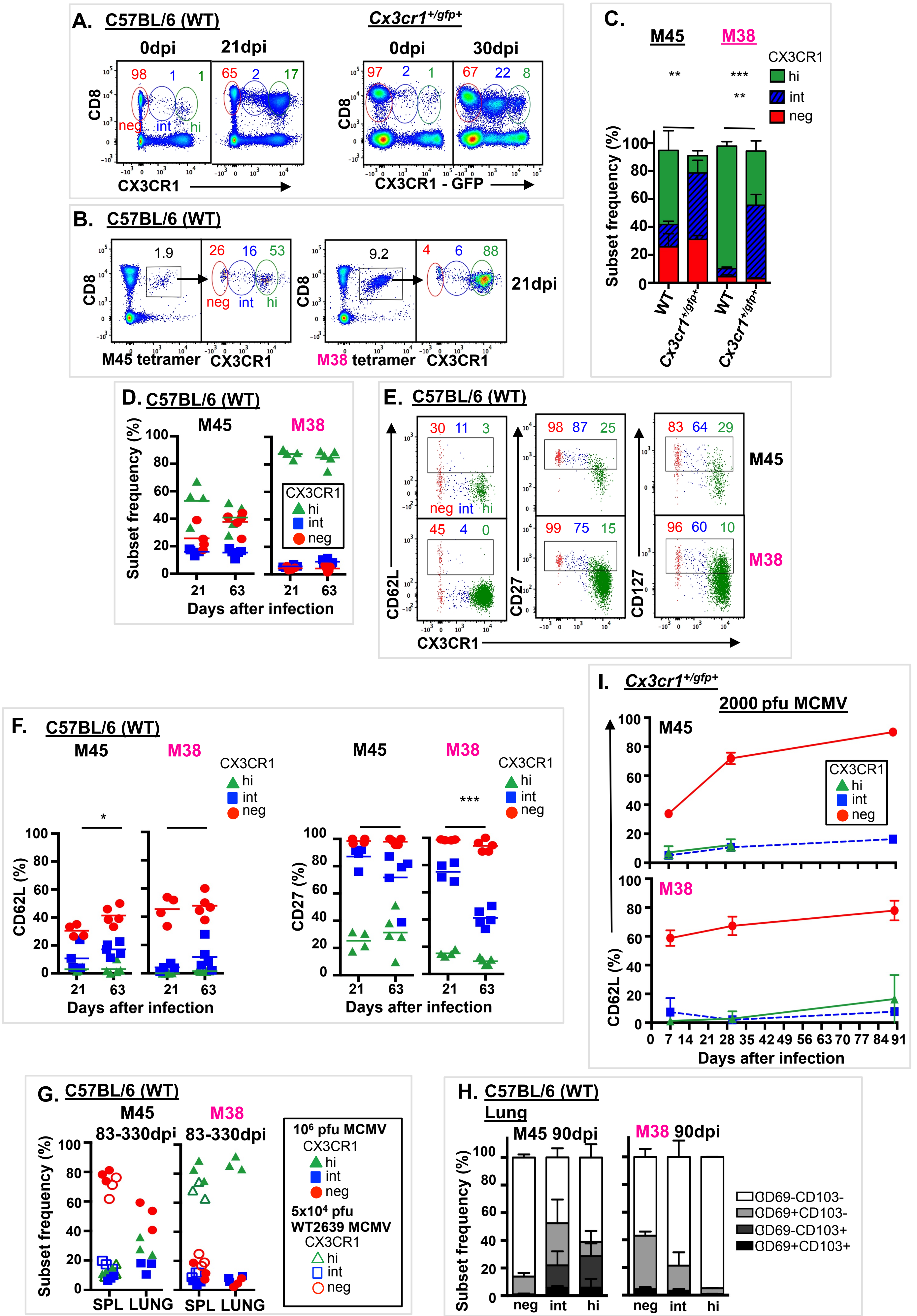

A. C57BL/6 (WT)
0dpi
21dpi
98 1 1
neg int hi
65 2 17
CD8
CX3CR1
Cx3cr1+/gfp+
0dpi
30dpi
97 2 1
67 22 8
CX3CR1 - GFP
B. C57BL/6 (WT)
1.9
26 16 53
neg int hi
M45 tetramer
CX3CR1
9.2
4 6 88
M38 tetramer
21dpi
C.
M45
M38
CX3CR1
hi
int
neg
Subset frequency (%)
WT
Cx3cr1+/gfp+
D. C57BL/6 (WT)
M45
M38
Subset frequency (%)
Days after infection
E. C57BL/6 (WT)
30 11 3
45 4 0
98 87 25
99 75 15
83 64 29
96 60 10
M45
M38
CD62L
CD27
CD127
CX3CR1
F. C57BL/6 (WT)
CD62L (%)
CD27 (%)
Days after infection
G. C57BL/6 (WT)
M45 83-330dpi
M38 83-330dpi
10⁶ pfu MCMV
5x10⁴ pfu WT2639 MCMV
SPL LUNG
Subset frequency (%)
H. C57BL/6 (WT)
Lung
M45 90dpi
M38 90dpi
CD69-CD103-
CD69+CD103-
CD69-CD103+
CD69+CD103+
I. Cx3cr1+/gfp+
2000 pfu MCMV
CD62L (%)
Days after infection

Figure S2: MCMV infection induces three distinct $CD8^+$ T cell populations based on CX3CR1 expression in C57BL/6 and *Cx3cr1*$^{+/gfp+}$ mice, related to Figure 1

Cx3cr1$^{+/gfp+}$ and C57BL/6 (wild type; WT) mice were infected intravenously (iv) with 10^6 pfu or 2000 pfu MCMV. Conventional memory and inflationary memory responses were assessed by M45- and M38-tetramer staining, respectively. (A) Composite FACS plots of CD8 and CX3CR1 staining of live lymphocytes in blood from C57BL/6 (left panel) or *Cx3cr1*$^{+/gfp+}$ (right panel) mice at 0 days post infection (dpi), 21dpi (C57BL/6) or 30 dpi (*Cx3cr1*$^{+/gfp+}$) with 10^6 pfu MCMV. Mean percentages of CX3CR1 subsets (negative[neg]=red, intermediate[int]=blue, high[hi]=green) of live $CD8^+$ T cells are indicated (C57BL/6 n=4 mice, *Cx3cr1*$^{+/gfp+}$ n=3 mice). (B) Composite FACS plots of M45- and M38-tetramer staining of live lymphocytes (left) and CX3CR1 subsets of tetramer-specific $CD8^+$ T cells (right) in blood from C57BL/6 mice, 21 dpi with 10^6 pfu MCMV. Mean live $tetramer^+$ $CD8^+$ T cells and mean CX3CR1 subsets are indicated (n=4). (C) Compiled results comparing mean (±SD) of CX3CR1 subsets (neg=red, int=blue, hi=green) of M45- and M38-$tetramer^+$ $CD8^+$ T cells in blood from C57BL/6 (WT) or *Cx3cr1*$^{+/gfp+}$ mice, 21 or 30 dpi with 10^6 pfu MCMV, respectively (C57BL/6 n=4 mice, *Cx3cr1*$^{+/gfp+}$ n=3 mice). (D) Individual CX3CR1 subset frequencies and means of M45- and M38-$tetramer^+$ $CD8^+$ T cells in blood from C57BL/6 (WT) mice 21 and 63 dpi (n=4 and n=5, respectively). (E-F) Expression of cell surface markers CD62L, CD27 and CD127 by CX3CR1 subsets in M45- and M38-$tetramer^+$ $CD8^+$ T cells in C57BL/6 mice 21 or 63 dpi with 10^6 pfu MCMV. (E) Composite FACS plots of CD62L (left panel), CD27 (middle panel) and CD127 (right panel) expression in M45- and M38-$tetramer^+$ $CD8^+$ T cells at 21 dpi. Mean expression of indicated marker for each CX3CR1 subset is shown (n=4). (F) Shown are individual and mean CX3CR1 subset expression of CD62L (left panel) and CD27 (right panel) in M45- and M38-$tetramer^+$ $CD8^+$ T cells in blood from C57BL/6 (WT) mice 21 and 63 dpi (n=4/each and n=5/each, respectively).(G) CX3CR1 subset distribution of M38- and M45- tetramer+ cells was assessed in spleen (SPL), liver (LIV) and lung of C57BL/6 (WT) mice following infection with 10^6 pfu (solid symbols, 330dpi) or 50000 pfu (open symbols, 83dpi) of WT2639 MCMV (considered to be a full dose). (H) Frequency of co-expression of CD69 and CD103 in M45- and M38-$tetramer^+$ $CD8^+$ T cells in lung from C57BL/6 mice 83dpi (n=3). (I) Compiled results showing mean (±SD) CD62L expression by CX3CR1 subsets in M45- and M38-$tetramer^+$ $CD8^+$ T cells following infection with 2000 pfu MCMV (n=5-9). Significant differences were determined by 1-way ANOVA and corrected for multiple comparisons (Holm-Sidak). P=0.05 to 0.011 (*), p=0.01 to 0. 001 (**) and p<0.001 (***). All FACS plots are composite plots generated by randomly selecting equal numbers of the cell population of interest from each subject.

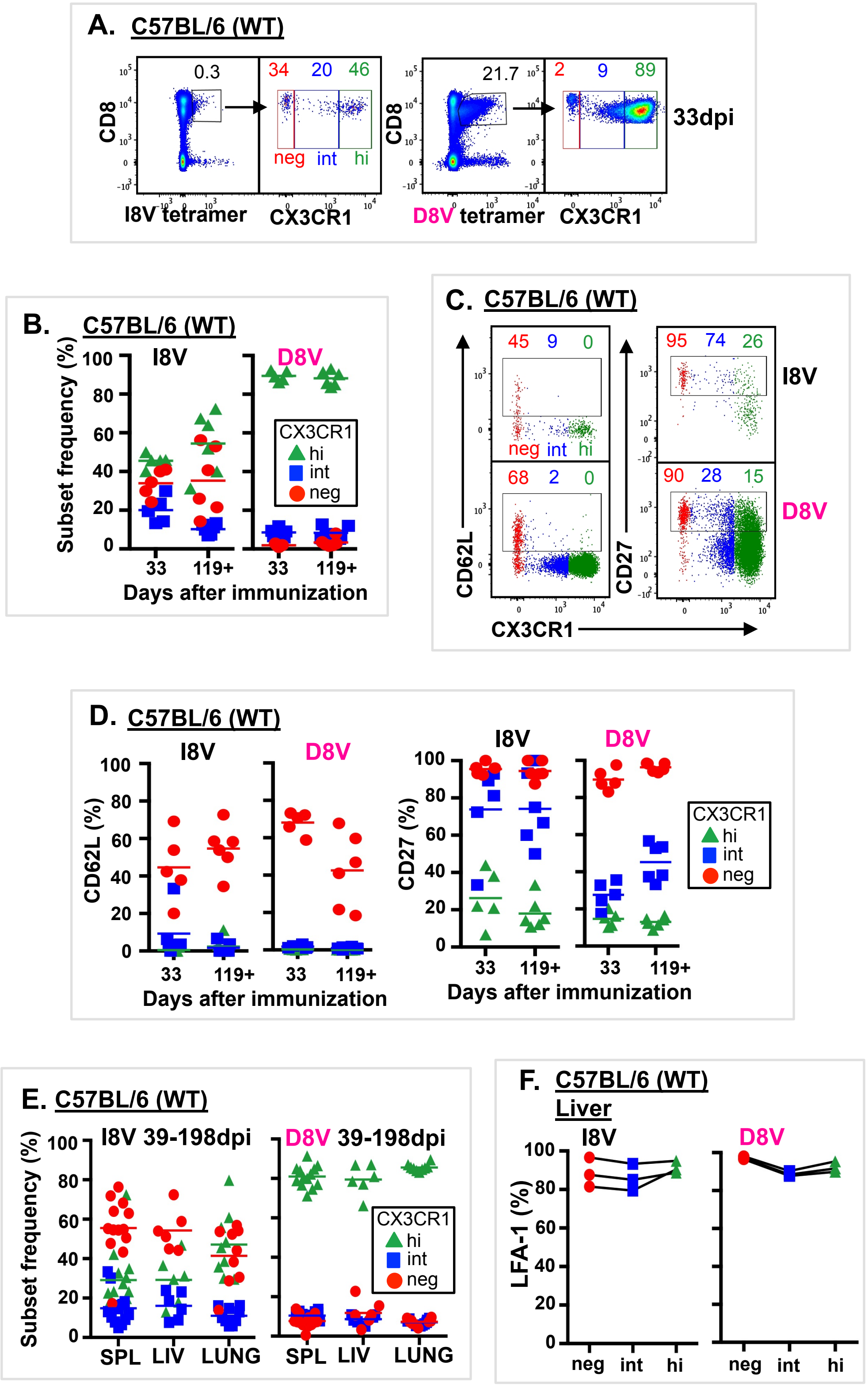
A. C57BL/6 (WT)
0.3
34 20 46
neg int hi
CD8
I8V tetramer
CX3CR1
21.7
2 9 89
D8V tetramer
CX3CR1
33dpi
B. C57BL/6 (WT)
I8V
D8V
Subset frequency (%)
CX3CR1
hi
int
neg
33 119+ 33 119+
Days after immunization
C. C57BL/6 (WT)
45 9 0
neg int hi
95 74 26
I8V
68 2 0
90 28 15
D8V
CD62L
CD27
CX3CR1
D. C57BL/6 (WT)
I8V
D8V
CD62L (%)
CD27 (%)
CX3CR1
hi
int
neg
Days after immunization
E. C57BL/6 (WT)
I8V 39-198dpi
D8V 39-198dpi
Subset frequency (%)
SPL LIV LUNG
CX3CR1
hi
int
neg
F. C57BL/6 (WT)
Liver
I8V
D8V
LFA-1 (%)
neg int hi

Figure S3: Ad-lacZ immunization induces three distinct CD8$^+$ T cell populations based on CX3CR1 expression in C57BL/6 mice, related to Figure 2

C57BL/6 (wild type; WT) mice were infected intravenously (iv) with 2x10^9 pfu of a recombinant replication-deficient HuAd5 vector expressing lacZ (Ad-lacZ). Conventional memory and inflationary memory responses were assessed by I8V- and D8V-tetramer staining, respectively. (A) Composite FACS plots of I8V- and D8V-tetramer staining of live lymphocytes (left) and CX3CR1 subsets of tetramer-specific CD8$^+$ T cells (right) in blood from C57BL/6 (WT) mice at 33 days post immunization (dpi) with 2x10^9 pfu Ad-lacZ. Mean CX3CR1 subsets (negative[neg]=red, intermediate[int]=blue, high[hi]=green) of live CD8$^+$ T cells are indicated (n=4 mice). (B) Shown are individual and mean CX3CR1 subset frequency of I8V- and D8V-tetramer$^+$ CD8$^+$ T cells in blood from C57BL/6 (WT) mice 33 and 119+ dpi (n=5 and n=6, respectively). (C-D) Expression of cell surface markers CD62L and CD27 by CX3CR1 subsets in I8V- and D8V-tetramer$^+$ CD8$^+$ T cells in C57BL/6 mice 33 or 119+ dpi with 2x10^9 pfu Ad-lacZ. (C) Shown is representative flow cytometry plots of CD62L (left panel) and CD27 (middle panel) expression in I8V- and D8V-tetramer$^+$ CD8$^+$ T cells at 33 dpi. Mean expression of indicated marker for each CX3CR1 subset is shown (n=5). (D) Individual and mean CX3CR1 subset expression of CD62L (left panel) and CD27 (right panel) in I8V- and D8V-tetramer$^+$ CD8$^+$ T cells in blood from C57BL/6 (WT) mice 33 and 119+ dpi (n=5 and n=6, respectively). (E) Individual and mean CX3CR1 subset frequencies of I8V- and D8V- tetramer+ cells in spleen (SPL), liver (LIV) and lung 39-198dpi (n=6-13). (F) Expression of LFA-1 in I8V- and D8V-tetramer$^+$ CD8$^+$ T cells in liver from C57BL/6 mice 198dpi (n=3). Significant differences were determined by one-way ANOVA and corrected for multiple comparisons (Holm-Sidak). P=0.05 to 0.011 (*), p=0.01 to 0. 001 (**) and p<0.001 (***). All FACS plots are composite plots generated by randomly selecting equal numbers of the cell population of interest from each subject.

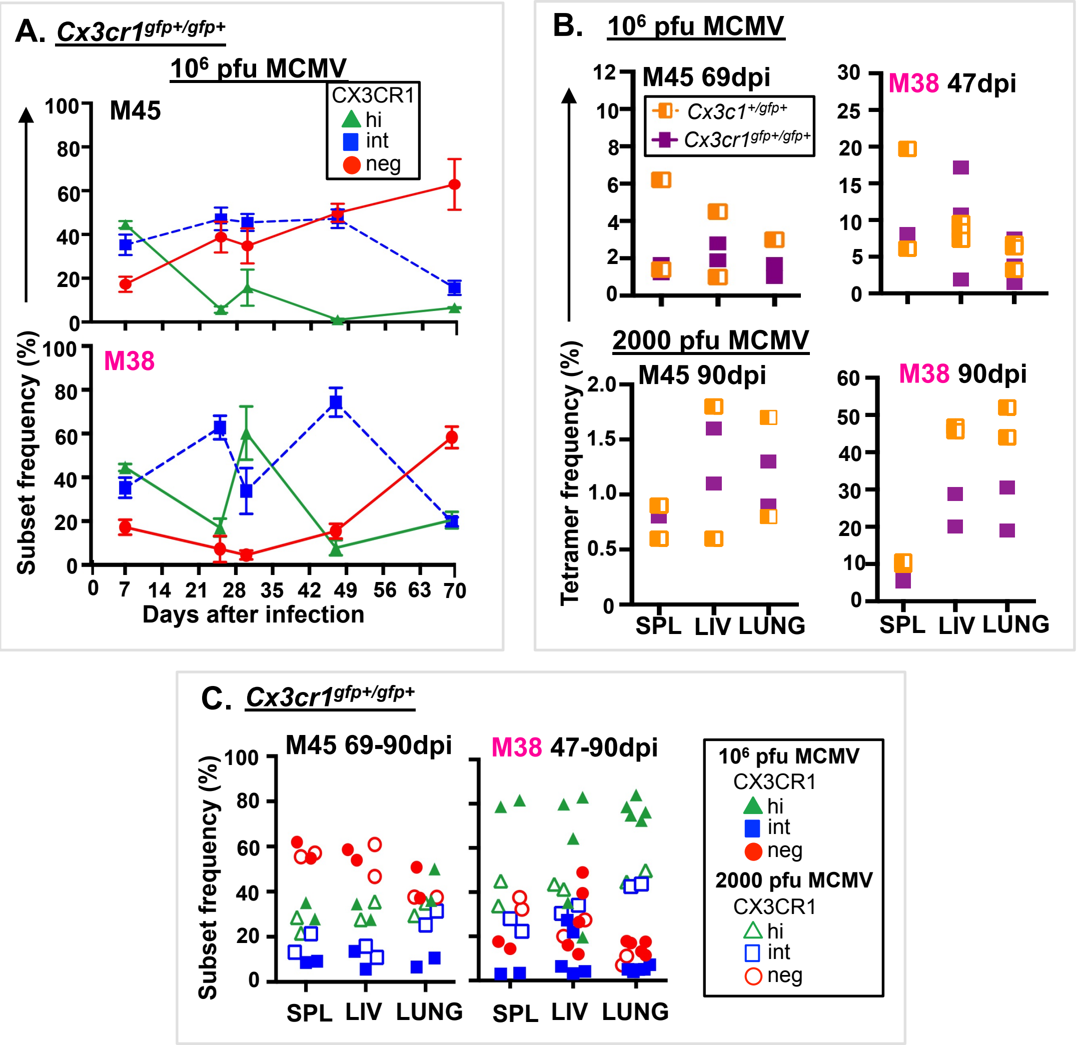
A. Cx3cr1gfp+/gfp+
10^6 pfu MCMV
M45
M38
CX3CR1
hi
int
neg
Subset frequency (%)
Days after infection
B. 10^6 pfu MCMV
M45 69dpi
Cx3c1+/gfp+
Cx3cr1gfp+/gfp+
M38 47dpi
2000 pfu MCMV
M45 90dpi
M38 90dpi
Tetramer frequency (%)
SPL
LIV
LUNG
C. Cx3cr1gfp+/gfp+
M45 69-90dpi
M38 47-90dpi
Subset frequency (%)
10^6 pfu MCMV
CX3CR1
hi
int
neg
2000 pfu MCMV
CX3CR1
hi
int
neg

Figure S4: CX3CR1 is not required for optimal inflating responses following MCMV infection, related to Figure 4

Cx3cr1$^{gfp+/gfp+}$ and *Cx3cr1*$^{+/gfp}$ mice were infected intravenously (iv) with 10^6 pfu or 2000 pfu MCMV and blood was serially sampled up to 70 days post-infection (dpi). Memory responses were assessed by M45 and M38 tetramer staining. (A) Compiled results showing mean (±SD) CX3CR1 subsets (neg=red circle, int=blue square, hi=green triangle) of M45- and M38-tetramer$^+$ CD8$^+$ T cells in *Cx3cr1*$^{gfp+/gfp+}$ mice following infection with 10^6 pfu MCMV (n=2-4 mice/each). (B) Individual tetramer frequencies of M45- and M38-tetramer$^+$ CD8$^+$ T cells in spleen (SPL), liver (LIV) and lung from *Cx3cr1*$^{+/gfp+}$ (orange half filled square, n=2-3 mice/each) and *Cx3cr1*$^{gfp+/gfp+}$ (purple filled square, n=3-4 mice/each) mice 47-90 dpi infection with 10^6 pfu (top panel) or 2000 pfu (bottom panel) MCMV. (C) Shown are individual CX3CR1 subset frequencies for M45- and M38-tetramer$^+$ CD8$^+$ T cells in indicated tissues, 47-90 dpi with 10^6 pfu (closed symbol) or 2000 pfu (open symbol) MCMV. Significant were determined by one-way ANOVA and corrected for multiple comparisons (Holm-Sidak). P=0.05 to 0.011 (*), p=0.01 to 0. 001 (**) and p<0.001 (***).

Figure S5

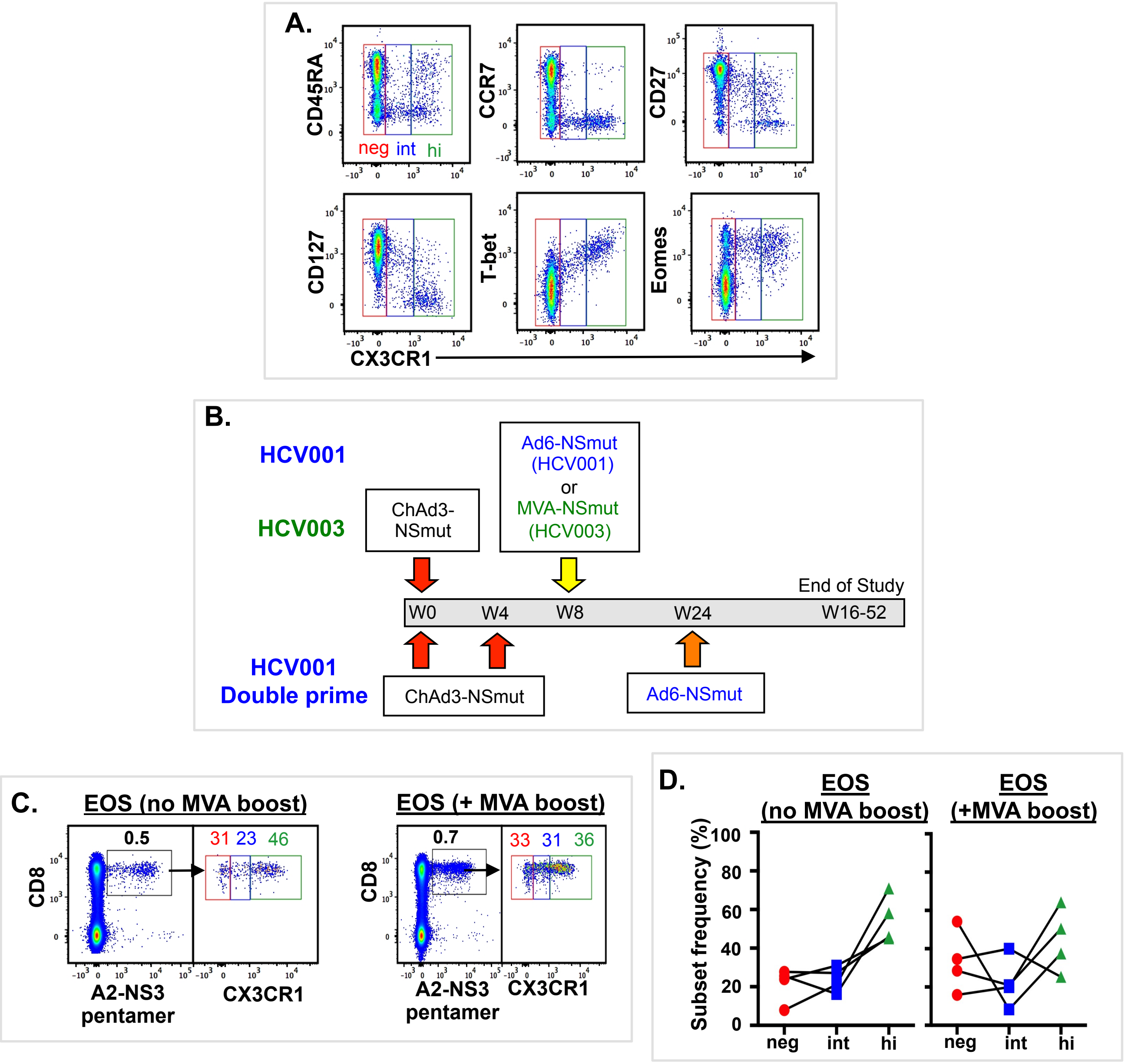
A.
CD45RA
CCR7
CD27
CD127
T-bet
Eomes
neg int hi
CX3CR1
B.
HCV001
HCV003
ChAd3-NSmut
Ad6-NSmut (HCV001) or MVA-NSmut (HCV003)
End of Study
W0 W4 W8 W24 W16-52
HCV001 Double prime
ChAd3-NSmut
Ad6-NSmut
C.
EOS (no MVA boost)
EOS (+ MVA boost)
0.5
31 23 46
0.7
33 31 36
CD8
A2-NS3 pentamer
CX3CR1
D.
EOS (no MVA boost)
EOS (+MVA boost)
Subset frequency (%)
neg int hi

Figure S5: Phenotype of CX3CR1 subsets on polyclonal and adenoviral vaccine-derived $CD8^+$ T cells, related to Figures 5-6

(A) Expression of cell surface markers (CD27 and CD127) and transcription factors (T-bet, Eomes) by CX3CR1 subset was assessed in A2-NS3 pentamer-negative $CD8^+$ T cells in blood taken from 5 volunteers. Shown are composite FACS plots (n=5) of CX3CR1 expression and indicated marker, gated on live CD8+ T cells with CX3CR1 subset highlighted (neg=red, int=blue, hi=green). (B) Schematic of vaccination protocol. All volunteers received a priming regimen of a replicative defective simian adenoviral vector (ChAd3, $5x10^8$ - $2.5x10^{10}$ viral particles) encoding the NS3, NS4, NS5A, and NS5B proteins of HCV genotype 1b (ChAd3-NSmut) (Swadling et al., 2014). A subset of 5 volunteers also received a boost with a modified vaccinia Ankara (MVA, $2x10^7$ - $2x10^8$ pfu) vector encoding the same HCV genotype 1b proteins (MVA-NSmut) 8 weeks (W) later (Table S2). (C) Blood samples were taken at end of study (TW16-32, "EOS") from all volunteers. Vaccine-derived $CD8^+$ T cells were assessed by staining with HLA-A2 pentamers containing an NS3 epitope (A2-NS3 pentamer; see Table S3 for pentamer details). Shown is composite FACS plots of A2-NS3 pentamer staining (left) of live $CD3^+$ lymphocytes, and CX3CR1 subsets of A2-NS3 $pentamer^+$ $CD8^+$ T cells at EOS in volunteers who did not receive the MVA boost (left, n=4) and those who did (right, n=5). Mean A2-NS3 $pentamer^+$ $CD8^+$ T cell frequency and mean CX3CR1 subset frequency is indicated. (D) Individual CX3CR1 subset frequency of A2-NS3 $pentamer^+$ $CD8^+$ T cells at EOS in volunteers who did not receive the MVA boost (left, n=4) and those who did (right, n=5).

Table S1: Number of $CD8^+$ T cells isolated from tissues of *$Cx3cr1^{+/gfp+\ and}$* *$Cx3cr1^{gfp+/gfp+}$* mice, related to Figure 4

	**Mean number of $CD8^+$ T cells (no. of mice)**		
**Tissue**	***$Cx3cr1^{+/gfp+}$***	***$Cx3cr1^{gfp+/gfp+}$***	**P value**
MCMV			
Spleen	$2.7x10^8$ (3)	$3.3x10^8$ (4)	0.59
Liver	$2.6x10^7$(3)	$7.6x10^6$ (4)	0.06
Lung	$1.7x10^7$ (3)	$2.9x10^7$ (4)	0.33
Ad-lacZ			
Spleen	$2.3x10^7$ (3)	$8.9x10^6$ (5)	0.13
Liver	$1.6x10^6$ (4)	$9.9x10^5$ (5)	0.12
Lung	$1.4x10^6$ (4)	$9.1x10^5$ (5)	0.36

Table S2: Characteristics of 13 volunteers used for this study, related to Figures 5-7 and Figure S5

Volunteer#	Age	Sex	CMV IgG	ChAd3 prime	MVA boost	HCV pentamer[a]	CMV tetramer[a]
**006**	29	M	-	+	-	A2-NS3	NA
**038**	25	F	+	+	-	A2-NS3	NA
**060**	24	F	-	+	-	A2-NS3	NA
**068**	27	F	-	+	-	A2-NS3	NA
**332**	21	M	-	+	+	A2-NS3	NA
**339**	37	F	+	+	+	A2-NS3	A2-pp65
**343**	31	M	-	+	+	A2-NS3	NA
**345**	21	F	+	+	+	A2-NS3	A2-pp65
**347**	26	M	+	+	+	A2-NS3	NA
**039**	44	M	+	NA	NA	NA	A2-pp65
**400**	28	M	+	NA	NA	NA	B7-pp65
**401**	30	F	+	NA	NA	NA	A2-pp65, A1-pp50
**403**	54	M	+	NA	NA	NA	A2-pp65

[a]See Table S3 for tetramer and pentamer details
Abbreviations: Male (M), female (F), not applicable (NA), positive (+), negative (-), hepatitis C virus (HCV),

Table S3: List of vaccine pentamers and CMV tetramers used for studies antigen-specific CD8+ T cells, related to Experimental Procedures

HLA type	Target-epitope	Amino acid sequence	Abbreviation	Source
Human				
A*0101	CMV-pp50 $_{245–253}$	VTEHDTLLY	A1-pp65	NIH Tetramer Facility[a]
A*0201	CMV-pp65 $_{495–503}$	NLVPMVATV	A2-pp65	NIH Tetramer Facility[a], Proimmune
B*0702	CMV-pp65 $_{417–426}$	TPRVTGGGAM	B7-pp65	NIH Tetramer Facility[a]
A*0201	HCV-NS3$_{1406-1415}$	KLSGLGINAV	A2-NS3	Proimmune
Mouse				
H-2Db	MCMV-M45$_{985-993}$	HGIRNASFI	M45	NIH Tetramer Facility[a]
H-2Kb	MCMV-M38$_{316-324}$	SSPPMFRV	M38	NIH Tetramer Facility[a]
H-2Kb	bgal$_{497-504}$	ICPMYARV	I8V	NIH Tetramer Facility
H-2Kb	bgal$_{96-103}$	DAPIYTNV	D8V	NIH Tetramer Facility[a]

[a]Peptide for monomer construction was obtained from Proimmune.

Table S4: Fluorochrome-conjugated antibodies used in flow cytometry, related to Experimental Procedures

Antibody	Fluorochrome	Clone	Manufacturer[a]
Human			
CD3	PO	UCHT1	Life Technologies
CD8	AF700	SK1	BioLegend
CCR7	PerCp-Cy5.5	G04H37	BioLegend
CD45RA	FITC	HI100	Beckton Dickinson
CX3CR1	BV421	2A9-1	BioLegend
CD27	PE-Cy7	LG.3A10	BioLegend
CD127	PE/Dazzle594	A019D5	BioLegend
T-bet	BV605	4B10	BioLegend
Eomes	eF660	WD1928	eBioscience
Viability	LIVE/DEAD near IR	NA	Life Technologies
Mouse			
CD8	eF450	53-6.7	eBioscience
CX3CR1	BV421	SA011F11	BioLegend
CD62L	AF700	MEL-14	BioLegend
CD27	FITC	LG.7F9	eBioscience
CD27	PerCp-Cy5.5	LG3A.10	BioLegend
CD127	PE-Cy7	A7R34	eBioscience
Thy1.2	AF700	53-2.1	Biolegend
Viability	LIVE/DEAD near IR	NA	Life Technologies

[a] Antibodies were obtained from BD Biosciences (Oxford, UK), eBioscience (Loughborough, UK), BioLegend (London, UK) and Life Technologies (Loughborough, UK.

Abbreviations: Pacific orange (PO), alexa Fluor (AF), allophycocyanin (APC), brilliant violet (BV), eFluor (eF), infrared (IR), peridinin-chlorophyll-protein (PerCP), phychoerythrin (PE) and fluorescein isothiocyanate (FITC).

Italics denote the species specificity of the antibody
